# Investigating small molecules in propolis as Nipah virus glycoprotein (NiV-G) inhibitors through molecular interaction studies

**DOI:** 10.1016/j.heliyon.2025.e42595

**Published:** 2025-02-10

**Authors:** Muaz Faruque, Md Afjalus Siraj, Md Nazmul Hasan Zilani, Asish Kumar Das, Md Anisuzzman, Md Monirul Islam

**Affiliations:** a Pharmacy Discipline, Life Science School, Khulna University, Khulna, 9208, Bangladesh; b Department of Pharmacy, Faculty of Health Sciences, Gono Bishwabidyalay, Dhaka, 1344, Bangladesh; c Department of Pharmacy, Jashore University of Science & Technology, Jashore, 7408, Bangladesh

**Keywords:** Propolis, Nipah virus (NiV), Nipah virus glycoprotein (NiV-G), Molecular dynamics simulation, MM-PBSA

## Abstract

Despite the significant fatality rates associated with Nipah virus (NiV) outbreaks in South Asia, including Bangladesh, and India, till today, there is no approved medications to treat it. In this context, small molecules in propolis were computationally screened through pharmacokinetic and toxicity studies followed by molecular docking and dynamics simulation with Nipah virus glycoprotein (NiV-G protein) to assess their anti-Nipah potential. A thorough literature analysis was performed to identify antiviral compounds in propolis from a pool of 84 experimental articles. Following ADMET analysis, 27 molecules out of 34 were docked against NiV-G and compared with a control ligand, ribavirin, which is an investigational drug against Nipah. The molecular docking revealed that bauer-7-en-3β-yl acetate (BA) and moronic acid (MA) bound more strongly to the active site of NiV-G than ribavirin and other ligands. Investigation of root-mean-square deviation (RMSD), root mean square fluctuations (RMSF), radius of gyration (Rg), solvent accessible surface area (SASA), molecular surface area (MolSA), binding free energy (MM-PBSA), the complexity of hydrogen bonds (HBs), and secondary structure of ligand-target interactions for 100 ns by molecular dynamics (MD) simulation study further supported the docked complex's stability and compactness. Therefore, the in silico molecular interaction analysis reports that both molecules may be the possible candidates against Nipah infection.

## Highlights

• NIPAH virus (NiV) is the most fatal emerging single-stranded RNA virus in the Henipavirus genus.
• Nipah virus accounts for 40–70 % of deaths in infected individuals.
• No vaccines or therapeutic medicines for NiV have been approved to date.
• This study comprises a thorough literature analysis that identifies 34 antiviral compounds in propolis, which were computationally screened to investigate their inhibitory potential against NiV.
• Molecular interaction indicated that bauer-7-en-3β-yl acetate (BA) and moronic acid (MA) may act as promising inhibitors of NiV-G protein.

## Teaser

NIPAH virus (NiV) is the most fatal emerging single-stranded RNA virus in the Henipavirus genus. NiV due to its high pathogenicity and mortality rate is considered as a Biosafety Level 4 (BSL4) pathogen. Since its first outburst in Malaysia in 1988, near-annual infections have been seen predominantly in Bangladesh, India, and Malaysia. NiV infections in humans cause fever, acute respiratory syndrome, encephalitis with severe neurological complications, and myocarditis in few cases. Nipah virus accounts for 40–70 % of death of infected individuals, naming it as a potential pandemic threat in future. However, no vaccines or effective treatment regimens have been licensed yet against NiV infection. It is therefore crucial to find out and develop anti-NiV therapeutic agents. Here we report the promise of small molecules in propolis as NiV glycoprotein inhibitors. The findings of this study found a ground for the future research towards the discovery of drugs against NiV.

## 1 Introduction

The world has witnessed new deadly viruses and their resurgence with heterogeneity periodically throughout history [1,2]. Swine flu, different types of coronas, Ebola, and Zika are among the most pronounced viruses that caused significant pandemics in the last two decades [3]. Surprisingly, the entire world halted and became unfit in 2019 with the surge of COVID-19, which has exacted 6.8 million lives worldwide since its inception [4]. Though the discovery of successful therapeutics, including vaccines in an unprecedentedly fast time frame by extensive efforts from academia and industry, helped to reverse the situation [5], this remains quite challenging when confronting outbreaks endemic only to specific areas or which hit only a handful number of patients [6]. The outbreak of the NIPAH virus is endemic that occurs in Southeast Asian countries almost every year, and the World Health Organization recognizes it as a priority pathogen, posing potential epidemic threats due to its high pathogenicity and mortality rates [7].

NIPAH virus (NiV) is the most fatal emerging single-stranded RNA virus in the Henipavirus genus [8]. NiV upsurge was first identified in Malaysia in the late 1990s, claiming a 40 % lethality rate. In 1999, outbreaks were reported in Singapore, where separate investigations identified 13 and 11 cases, as diagnosed [9,10]. Soon after, NIPAH infections were reported in India and Bangladesh in 2001 [11]. Since then, sporadic NiV outbreaks have been observed annually in Bangladesh, especially during winter, and have engendered 72 % of the deaths of infected individuals till 2021 [12]. Even in January and February of 2023, 8 out of 11 reported NiV cases died throughout the country [13]. Meanwhile in Philippines, a 2014 outbreak investigation identified 17 positive Nipah virus cases (9 were dead) highlighting the ongoing risk of outbreaks in the region [14].

NiV primarily spreads zoonotically, but human-to-human transmission also happens and was first found in India and Bangladesh. *Pteropus* spp. fruit bats and pigs are natural reseroirs of it [15]. NiV infects the host cell through G protein, which underscores its significance as a potential therapeutic target. NiV, a negative-sense RNA virus, with a genome of ∼18,250 nucleotides, encodes six structural (N, P, M, F, G, and L) and three non-structural proteins (V, W, and C) [[16], [17], [18]]. These proteins orchestrate viral replication, immune evasion, and pathogenesis. Notably, the G protein contributes to viral attachment by binding to ephrin-B2 and B3 receptors, which are predominantly expressed on endothelial cells, neurons, and arterial smooth muscle [[19], [20], [21]]. Subsequent fusion of viral and host cell membranes, mediated by the F protein, enables viral entry and infection [[22], [23], [24]]. NiV infection manifests complications of the cardiovascular and respiratory systems followed by acute encephalitis [25,26], and supportive care for proper breathing and circulation maintenance is the only treatment modality to manage the illnesses [27]. Previously, favipiravir, acyclovir, and ribavirin were employed to combat and control the infection in the emergency state of febrile encephalitis, but these are not approved yet as anti-Nipah therapeutics [28,29]. The lack of effective therapeutics, thus, calls for robust efforts from all over the globe to develop drugs against NiV that has driven diverse approaches, including identification of small-molecule inhibitors through computational strategies, development of vaccines, and monoclonal antibodies [24,26,27].

Computational strategies utilize advanced techniques and tools to discover and design new molecules that can serve as starting points (lead compounds) for drug development by targeting specific biological molecules (e.g., proteins or receptors). Compared to conventional drug discovery techniques, computational methods offer a faster and more cost-effective approach to identifying potential drug candidates. Researchers can screen vast libraries of compounds in a short time and predict their binding affinities to key biological targets by employing molecular modeling, docking, and dynamics simulations. This approach facilitates the identification of promising candidates before in vitro or in vivo validation [30,31]. For these reasons, we adopted a computational strategy targeting the NiV-G protein for anti-Nipah drug development, incorporating in silico methods such as molecular docking and dynamic simulations.

Propolis, or bee glue, is a complex admixture of compounds that is a naturally occurring resinous product [32]. When the resinous and glue-like properties of propolis were put to use, it quickly made its way into traditional medicine cures [33]. Propolis's resin is gathered from leaf buds and crevices in tree bark by honeybees (*Apis mellifera* L.) to polish interior walls and fill gaps in their honeycombs [34]. Propolis has been utilized by humans for a very long time, with its earliest recorded application focusing on its antibacterial properties [35]. In sensitivity tests, propolis's extract exhibited a reduction in the quantity of *Candida* on 80 strains of *Candida* yeast [36]. Moreover, a study revealed a synergistic effect of propolis's ethanolic extract and anti-tuberculosis medications on mycobacteria development [37]. Furthermore, histopathological analysis revealed that it may be applied to treat bacterial nephropathy [38]. In addition, for the treatment of insulin-resistant diabetes, propolis has the potential to act as an antidiabetic agent [39]. Interestingly, some studies have suggested that propolis may have anti-cancer properties. For example, a study published in Evidence-Based Complementary and Alternative Medicine found that propolis reduced the growth of breast cancer cells in vitro [40]. It has also been demonstrated that propolis possesses antiviral activities against reoviruses, poliovirus, HIV, as well as influenza A and B viruses [41]. The unique properties and diverse range of therapeutic effects of propolis are now being regarded as a functional ingredient of food products by researchers [42]. Given these attributes, this study aims to investigate the inhibitory potential of compounds in propolis against the NiV G protein using in silico methods, focusing on disrupting its interaction with ephrin-B2 and B3 receptors. By targeting the active sites of the G protein (Gln559, Glu579, Tyr581, and Ile588) [43], the present approach seeks to explore new therapeutic avenues for combating NiV infection.

## 2 Methods

### 2.1 Data sources and searches

For evaluation of the antiviral activity, articles published in Google Scholar, PubMed, ScienceDirect, JSTOR, and ProQuest were searched. The keywords for searching the articles were “propolis” + “antiviral” + “activity” + “isolated compounds” + “compounds”. The searched articles were from 1985 to 2023 (June) based on in vivo and in vitro studies. Review articles were exempted, and no compounds were taken from articles based on in silico studies. Finally, 84 experimental articles were chosen to find out the antiviral compounds of propolis following the identification of duplicate articles. The 2D structures of chosen antiviral compounds are listed in the Supplementary Fig. 1 along with their in vivo and in vitro antiviral activity in Table 1.

**Table 1:** Antiviral compounds in propolis.

Compound	Effect	Reference
Quercetin	Anti-coronavirus, anti-human rhinoviruses (HRVs)	[44,45]
Rutin	Anti-coronavirus	[44]
Caffeic acid	Anti-coronavirus	[44]
Acacetin	Anti-HRV-2, HRV-3, and HRV-4	[45]
Chrysin	Anti-coronavirus	[45,46]
Kaempferol	Anti-coronavirus, anti-HRVs, and in vivo anti-influenza	[44,45,47]
p-Coumaric acid	In vitro anti-HRVs, anti-herpes simplex virus (HSV) type 1	[45,48]
Ferulic acid	Anti-porcine parvovirus	[49,50]
Isopentyl ferulate	In vivo anti-influenza A (H3N2)	[47]
Apigenin	In vivo anti-influenza (H1N1)	[47]
3,4-dicaffeoylquinic acid	Anti-influenza	[51,52]
Moronic acid	Anti-HIV, Anti-epstein–barr virus	[53,54]
Isorhamnetin	Anti-HIV	[55]
Diprenylcinnamic acid	Anti-HIV	[55]
Naringenin	Anti-HIV	[55]
Galangin	Anti-HRV, anti-HSV-1	[45,48]
12-Acetoxytremetone	Anti-HIV	[56]
Fisetin	Anti-HRV-2, HRV-3, and HRV-4	[45]
Luteolin	Anti-HRV-2, HRV-3, and HRV-4	[45]
Caffeic acid phenethyl ester	Anti-HSV	[57]
Cinnamic acid	Anti-poliovirus type 1 (PV1)	[58]
3-methyl-but-2-enyl caffeate	Anti-HSV	[59]
Medicarpin	Anti-Chikungunya virus (CHIKV)	[60]
Neovestitol	Anti- CHIKV	[60]
Vestitol	Anti- CHIKV	[60]
Plukenetione A	Anti-lentiviral activity	[61]
Pinostrobin	Anti-coronavirus	[62]
Bauer-7-en-3β-yl acetate	Anti-pseudorabies virus	[63]
3,5-Dicaffeoylquinic acid	Anti-influenza	[51]
4,5-Dicaffeoylquinic acid	Anti-influenza	[51]
3,4,5-Tricaffeoylquinic acid	Anti-influenza	[51]
Chlorogenic acid	Anti-influenza	[51]
Pinocembrin	Anti-coronavirus	[64]
Hesperetin	Anti-coronavirus	[64]

### 2.2 Toxicity profile from ADMET analysis

The SwissADME [65] and pkCSM [66] websites were used to evaluate the compounds' pharmacological and toxicological effects. Based on the Lipinski filter rule, a compound's drug-likeness is regarded acceptable with molecular weights not more than 500 g/mol, Log P not greater than 5, H-bond donors not greater than 5, and H-bond acceptors not greater than 10 [67]. Compounds with more than two violations were not analyzed for further experiments.

### 2.3 Protein preparation

The 3D structure of the Nipah virus attachment glycoprotein (NiV-G) was extracted from the protein data bank (PDB ID: 2VSM) [68]. This glycoprotein was structurally reported by X-ray diffraction at a high resolution of 1.80 Å as a hydrolase complexed with the human cell surface receptor ephrin-B2, and had no known mutations involved in this complex. The quality of the crystallographic model was indicated by important experimental numbers, such as an R-Value Free of 0.198, an R-Value Work of 0.152, and an R-Value observed of 0.154. To start the cleaning procedure of protein, the natural ligands and water molecules were exterminated. Only NiV-G protein chain A was selected for analysis. Before performing energy minimization, the hydrogen molecules were added by YASARA software [69]. Amino acid residues Gln559, Glu579, Tyr581, and Ile588 were chosen as the active site [43].

### 2.4 Ligand preparation

PubChem database was utilized for downloading the SDF format of ligands [70]. If any of the ligand's SDF format was not available on PubChem, the structure was constructed through ChemSketch software [71]. Finally, energy minimization was done by using YASARA software [72].

### 2.5 Protein-ligand binding analysis by molecular docking

The grid box with specific dimensions for x = 34.99, y = 34.99, z = 34.99 Å; α = β = γ = 90° was positioned surrounding NiV-G (PDB ID: 2VSM) active site which consists of the amino acid residues Gln559, Glu579, Tyr581, and Ile588. Interaction analysis through molecular docking was executed by using the YASARA macro dock_run.mcr, on the YASARA software (Version 22.8.22.W.64) [72] by clustering 25 vina dockings [73]. The force field was selected as default (AMBER14). The Gibbs free energy (ΔG, kcal/mol) was calculated using the YASARA software; higher positive energy values suggest stronger interaction [69]. The energy-based scoring mechanism that comes with YASARA by default was used to score the data. Following docking, the top-ranked positions were examined according to their closeness to important binding site residues, binding energy, and ligand-receptor interactions. Using the AMBER force field and energy minimization, the optimal position was further optimized. Docking results were stored in CSV format for statistical analysis and PDB format for structural visualization. YASARA software's [72] align feature was used for superimposing protein-ligand complex structures [74]. The interactive residues, distances, and bonds were visualized by the BIOVIA Discovery Studio 2021 Client [75].

### 2.6 Molecular dynamics (MD) simulation for 100 ns

Molecular dynamics (MD) simulations were performed using YASARA Dynamics with the AMBER14 force field across various version (v22.8.22.W.64) [72]. A periodic simulation cell was used, providing a buffer of 15 Å around the protein complex, filled with water at a density of 0.997 g/mL. Cl⁻ ions were added to neutralize the system [76], maintaining physiological conditions with a total NaCl concentration of 0.9 % and pH adjusted to 7.4. Hydrogen atoms were added to ionizable groups based on their pKa values computed using the Ewald method. Energy minimization was performed using the steepest descent method under relaxed constraints. Under constant pressure-constant temperature (NPT) conditions, simulations were carried out at 25 °C (298 K) and 1 atm with a timestep of 2.25 fs for a total of 100 ns. For short-range van der Waals and Coulomb interactions [77], a cutoff radius of 8 Å was used. RMSD, RMSF, radius of gyration (Rg), solvent-accessible surface area (SASA), molecular surface area (MolSA), MM-PBSA, and hydrogen bond counts were among the trajectory analyses and data were gathered at 100 ps intervals. To guarantee consistency and reproducibility, a pre-installed macro (md_run.mcr) was used to carry out each step.

### 2.7 Binding free energy analysis from MM-PBSA calculation

Over the course of 100 ns, MD simulation data of investigational complexes were gathered every 100 ps. Using the YASARA macro md_analyze bind energy.mcr, the MM-PBSA calculations of solvation binding energy were performed, with more positive values indicating more stability [78]. The following equation was used to represent the binding free energy (kcal/mol):

ΔE_binding_ = [potential energy for the free protein(j) + solvation energy for the free protein(j) + potential energy for the free ligand + solvation energy for the free ligand] - [potential energy for the complex(j) + solvation energy for the complex(j)]

Where, j is the position number.

## 3 Results

### 3.1 Pharmacokinetics and toxicity profile

Literature searches of the propolis's compounds yielded 34 antiviral chemicals (Table 2), which were studied according to their drug-likeness and safety profile. Ligands that did not pass the Lipnkski rule are rutin, 3,4-dicaffeoylquinic acid, 3,5-dicaffeoylquinic acid, 4,5-dicaffeoylquinic acid, and 3,4,5-tricaffeoylquinic acid, and were excluded for further study. 12-acetoxytremetone, vestitol were also discarded because they exhibited AMES toxicity. And hepatoxicity was measured in pinostrobin.

**Table 2:** Pharmacokinetics and toxicity profile.

Compounds	MW (g/mol)	LogP	NHA	NHD	NRB	MR	TPSA (Å^2^)	BBBP	IA	TC	LD_50_	MTD	HT	AT	NLV	DL
Quercetin	302.24	1.990	7	5	1	78.03	131.36	No	77.207	0.407	2.471	0.499	No	No	0	Yes
Rutin	610.52	−1.69	16	10	6	141.38	269.43	No	23.45	−0.369	2.491	0.452	No	No	3	No
Caffeic acid	180.16	1.195	4	3	2	47.16	77.76	No	69.407	0.508	2.383	1.145	No	No	0	Yes
Chrysin	254.24	2.871	4	2	1	71.97	70.67	Yes	93.76	0.405	2.289	0.016	No	No	0	Yes
Kaempferol	286.24	2.282	6	4	1	76.01	111.13	No	74.29	0.477	2.449	0.531	No	No	0	Yes
p-Coumaric acid	164.16	1.490	3	2	2	45.13	57.53	Yes	93.494	0.662	2.155	1.111	No	No	0	Yes
Ferulic acid	194.18	1.4986	4	2	3	51.63	66.76	Yes	93.685	0.623	2.282	1.082	No	No	0	Yes
Isopentyl ferulate	264.32	3.000	4	1	7	75.18	55.76	Yes	92.688	0.644	1.978	0.63	No	No	0	Yes
Apigenin	270.24	2.577	5	2	1	73.99	90.90	No	93.25	0.566	2.45	0.328	No	No	0	Yes
3,4-dicaffeoylquinic acid	516.45	10.296	12	7	9	126.90	211.28	No	29.037	−0.042	2.626	0.393	No	No	3	No
Moronic acid	454.68	7.442	3	1	1	135.69	54.37	No	100	−0.117	2.26	0.337	No	No	1	Yes
Isorhamnetin	316.26	2.291	7	4	2	82.50	120.36	No	76.014	0.508	2.407	0.576	No	No	0	Yes
Diprenylcinnamic acid	284.39	5.123	2	1	6	92.14	37.30	Yes	91.915	0.858	3.126	−1.164	No	No	1	Yes
Naringenin	272.25	2.509	5	3	1	71.57	86.99	No	91.31	0.06	1.791	−0.176	No	No	0	Yes
Galangin	270.24	2.576	5	3	1	73.99	90.90	No	93.985	0.256	2.45	0.333	No	No	0	Yes
12-Acetoxytremetone	260.29	2.312	4	0	5	70.83	52.60	Yes	96.587	0.485	1.944	0.588	No	Yes	0	Yes
Fisetin	286.24	2.282	6	4	1	76.01	111.13	No	83.752	0.421	2.465	0.579	No	No	0	Yes
Luteolin	286.23	2.282	6	4	1	76.01	111.13	No	81.13	0.495	2.455	0.499	No	No	0	Yes
Caffeic acid phenethyl ester	284.31	2.896	4	2	6	80.77	66.76	Yes	90.637	0.547	2.12	−0.121	No	No	0	Yes
Cinnamic acid	148.16	1.784	2	1	2	43.11	37.30	Yes	94.833	0.781	2.094	1.11	No	No	0	Yes
3-methyl-but-2-enyl caffeate	248.27	2.620	4	2	5	70.23	66.76	Yes	89.669	0.554	1.933	0.605	No	No	0	Yes
Medicarpin	270.28	3.010	4	1	1	73.17	47.92	Yes	95.188	0.273	2.512	−0.102	No	No	0	Yes
Neovestitol	272.30	2.825	4	2	2	75.62	58.92	Yes	93.896	0.31	2.218	−0.246	No	No	0	Yes
Vestitol	272.30	2.825	4	2	2	75.62	58.92	Yes	92.26	0.272	2.077	0.149	No	Yes	0	Yes
Plukenetione A	500.67	6.904	4	0	7	148.61	68.28	No	100	0.352	1.831	0.352	No	No	1	Yes
Pinostrobin	270.28	3.107	4	1	2	74.02	55.76	Yes	93.762	0.236	2.152	0.260	Yes	No	0	Yes
Bauer-7-en-3 β -yl acetate	468.75	8.595	2	0	2	144.88	26.30	No	98.981	0.022	1.971	0.051	No	No	1	Yes
3,5-Dicaffeoylquinic acid	516.45	1.029	12	7	9	126.90	211.28	No	44.225	−0.044	2.643	0.393	No	No	3	No
4,5-Dicaffeoylquinic acid	516.45	1.029	12	7	9	126.90	211.28	No	29.037	−0.042	2.626	0.393	No	No	3	No
3,4,5-Tricaffeoylquinic acid	678.59	2.705	15	8	13	170.29	257.81	No	41.915	−0.203	2.511	0.45	No	No	3	No
Chlorogenic acid	354.31	−0.645	9	6	5	83.50	164.75	No	36.377	0.307	1.973	−0.134	No	No	1	Yes
Pinocembrin	256.25	2.804	4	2	1	69.55	68.76	Yes	92.417	0.122	1.586	0.269	No	No	0	Yes
Hesperetin	302.28	2.518	6	3	2	78.06	96.22	No	70.277	0.044	2.042	0.25	No	No	0	Yes
Acacetin	284.26	2.879	5	2	2	78.48	79.90	No	94.318	0.663	2.22	0.09	No	No	0	Yes

Here, MW: molecular weight; NRB: no. of rotatable bonds; NHA: no. of hydrogen bond acceptor; NHD: no. of hydrogen bond donor; MR: molar refractivity (40–130); TPSA: topological polar surface area (less than or equal 140 Å^2^); logP: predicted octanol/water partition coefficient (<4.5); BBBP: blood-brain barrier permeability; IA: intestinal absorption (% absorbed); TC: total clearance (log ml/min/kg); LD_50_: oral rat acute toxicity; MTD: maximum tolerated dose for human (log mg/kg/day); HT: hepatotoxicity; AT: AMES toxicity; NLV: number of Lipinski's violation; DL: drug-likeness.

### 3.2 Protein-ligand binding studies

**Fig. 1:**
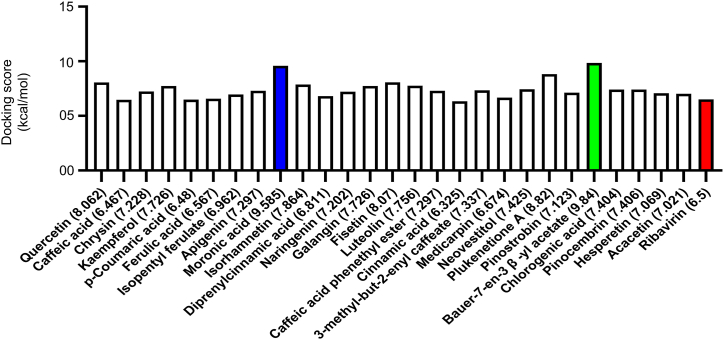
Docking scores between the NiV-G protein and 27 antiviral drugs generated from propolis and the control medication ribavirin. Each compound's Gibbs free energy (ΔG, kcal/mol) is displayed in the chart according to 25 VINA docking clusters. Moronic acid (MA) scored 9.585 kcal/mol, whereas Bauer-7-en-3β-yl acetate (BA) scored the most at **9.84** kcal/mol. With a score of **6.5** kcal/mol, the control medication ribavirin demonstrated a lower binding affinity than BA and MA. YASARA-generated higher positive score suggests the least binding free energy.

**Fig. 2:**
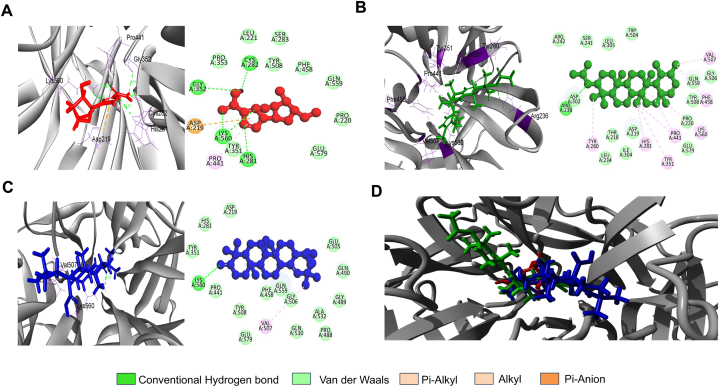
Results of Molecular Docking for 2VSM-Ligand Complexes_**A**. The greatest binding affinity (**9.84 kcal/mol**) is exhibited by the 2VSM–bauer-7-en-3β-yl acetate (BA) complex, which interacts with important amino acid residues and forms stable hydrogen bonds. **B**. With a score of **9.585** kcal/mol, the 2VSM–moronic acid (MA) complex shows somewhat lower binding than BA, most likely because it has fewer hydrogen bonds. However, because of its pentacyclic shape, it remains stable **C**. With weaker hydrogen bonds and less advantageous binding, the 2VSM–ribavirin complex exhibits a significantly lower score (**6.5** kcal/mol) **D**. The superimposed map illustrates the structural advantages of BA and MA's pentacyclic frameworks by highlighting their superior binding sites when compared to ribavirin.

The most important step in computer-aided drug design (CADD) is protein-ligand binding through molecular docking [79]. Molecular docking was conducted on NiV-G protein with 27 propolis's antiviral compounds and the control drug, ribavirin. 25 VINA docking clusters with the ligand to the receptor showed the following results in Fig. 1. Based on the cluster, the best interaction score (9.84 kcal/mol) was observed for bauer-7-en-3β-yl acetate (2VSM–BA complex), and the second best (9.585 kcal/mol) was seen at the 2VSM-moronic acid complex (2VSM-MA). On the other hand, 2VSM-ribavarin complex's score was 6.5 kcal/mol. The interacting amino acid residues of the three compounds are shown in Fig. 2(A–C) and Table 3. In addition, superimposed plot (Fig. 2D) of the complexes of Ribavirin, BA, and MA illustrated the bound position with the ligands.

**Table 3:** Non-bond interaction of the docked complexes.

Compound	Docking score (kcal/mol)	Interacting Residue	Distance (Å)	Bond Type
2VSM-MA	9.585	LYS560	2.24626	CH
		VAL507	5.09979	A
2VSM-BA	9.84	ARG236	2.51835	CH
		ARG236	2.20526	CH
		PRO441	5.4927	A
		LYS560	3.75736	A
		VAL507	3.69448	A
		PRO441	4.46824	A
		TYR280	4.65787	PA
		HIS281	4.60628	PA
		TYR351	4.97201	PA
		PHE458	5.49219	PA
2VSM-Ribavirin	6.50	HIS281	2.22991	CH
		LYS560	2.14883	CH
		LYS560	2.17942	CH
		CYS282	2.01147	CH
		GLY352	2.60898	CH
		ASP219	4.26743	Pi-Anion
		PRO441	4.29154	PA
		HIS281	2.22991	CH
		LYS560	2.14883	CH
		LYS560	2.17942	CH

YASARA-generated higher positive score suggests the least binding free energy. Here, Conventional Hydrogen Bond = CH, Alkyl = A, Pi-Anion = PA, Pi-Alkyl = PA.

**Fig. 3:**
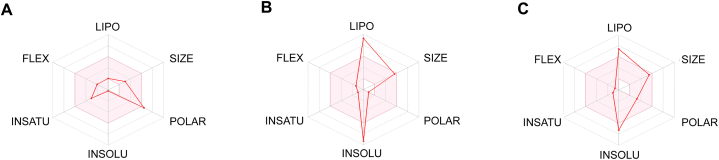
Physicochemical Properties Radar Charts for **A**. ribavirin, **B**. BA, and **C**. MA. SwissADME-generated radar charts show six important physicochemical properties, with the pink zone indicating their ideal ranges: size, lipophilicity (XLOGP3 between −0.7 and + 5.0), saturation (≥0.25 sp3 hybridized carbons), polarity (TPSA between 20 and 130 Å^2^), flexibility (≤9 rotatable bonds), and solubility (log S ≤ 0). BA and MA both match four of the six qualities, indicating that they are still suitable as drug-like options while being somewhat less ideal than ribavirin, which falls inside the optimal range for five of them.

We further utilized radar charts generated from the SwissADME website [Fig. 3(A-C)] to visually represent six critical physicochemical characteristics, offering insight into the distribution of key properties within the body. The pink zone within the charts denotes optimal ranges for each property, including polarity (POLAR) with TPSA between 20 and 130 Å^2^, size (SIZE), lipophilicity (LIPO) denoting XLOGP3 between −0.7 and + 5.0, solubility (INSOLU) with log S not exceeding 0, saturation (INSATU), where the proportion of carbons in the sp^3^ hybridization is not less than 0.25, and flexibility (FLEX), which is restricted to no more than nine rotatable bonds.

Ribavirin exhibited five characteristics falling within the acceptable zone. Conversely, both BA and MA demonstrated four out of six properties within the pink region, affirming their optimal suitability and compliance as drug-like compounds.

### 3.3 100 ns molecular dynamics simulation

Molecular docking studies does not take the dynamic behavior of protein molecules into account [80]. 100 ns MD simulations were performed to further validate the docking results and also analyze the docking complexes in an environment that is identical to human physiology.

#### 3.3.1 Root mean square deviation (RMSD)

**Fig. 4:**
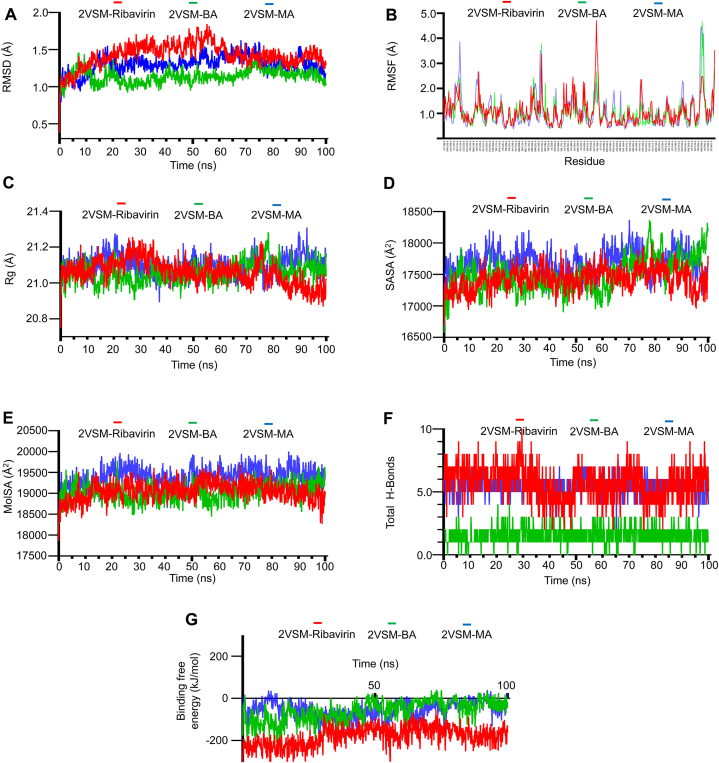
Simulation Analysis of 2VSM-Ligand Complexes Using Molecular Dynamics. **A**. According to RMSD analysis, the 2VSM-Ribavirin complex fluctuated the most (1.42 Å), followed by 2VSM-BA (1.14 Å) and 2VSM-MA (1.30 Å). **B**. According to the RMSF study, 2VSM-Ribavirin was more flexible (4.71 Å) than 2VSM-BA (4.65 Å) and 2VSM-MA (4.48 Å). **C**. In contrast to 2VSM-Ribavirin (21.06 Å) and 2VSM-MA (21.09 Å), 2VSM-BA was the most compact, according to the Rg values. **D**. The lowest value (17428.1 Å^2^) for 2VSM-Ribavirin was found by SASA, indicating improved accessibility for interaction in BA and MA. **E**. The highest surface interaction was found in 2VSM-MA (19384 Å^2^), followed by 2VSM-Ribavirin (19055 Å^2^) and 2VSM-BA (19035 Å^2^), according to MolSA values. **F**. The hydrogen bond study revealed that 2VSM-BA (∼2) and 2VSM-MA (∼5) generated fewer hydrogen bonds than 2VSM-Ribavirin (∼6). **G**. In contrast to 2VSM-Ribavirin (−180.52 kJ/mol), 2VSM-BA and 2VSM-MA showed higher binding energies (−60.08 and −53.13 kJ/mol, respectively), indicating superior stability, according to the Binding Free Energy study.

When estimating the conformational stability of proteins and protein-ligand complexes over time, RMSD is a crucial tool. After running simulation for 100 ns, average RMSD value of the 2VSM-Ribavirin complex (1.42 Å) was greater than the 2VSM-BA complex (1.14 Å) and 2VSM-MA complex (1.30 Å) shown in Fig. 4A. The 2VSM-Ribavirin complex expressed an increasing trend at 53.3 ns, and after that, the value decreased to 1.3 Å at 64.3 ns and was stable at 1.3 Å for the remaining period of the simulation. The complex of MA increased to 1.3 Å of RMSD after 19.2 ns and remained consistent for the next 60 ns, but at this point and also at 75 ns the value fluctuated. On the other hand, the 2VSM-BA complex showed deviation up to 20 ns and a slight increase at 73 ns, however it was stabilized for the rest of the simulation.

#### 3.3.2 Root mean square fluctuation (RMSF)

RMSF value describes the level of amino acid residues fluctuation and flexibility that a protein-ligand interface undergoes. The RMSF analysis (Fig. 4B) revealed that 2VSM-MA fluctuated at amino acid residues of 211–215, 335–336, 580–590 position and the complex of BA showed flexibility at 338–339, 580–590 amino acid residues. Similarly, the control drug complex, 2VSM-Ribavirin, showed less rigidity at 338–339, and 415–427 position. The fluctuation of the intense peak (4.71 Å) of the 2VSM-Ribavirin complex was greater than that of both ligand complexes, 2VSM-BA (4.65 Å) and 2VSM-MA (4.48 Å).

#### 3.3.3 Radius of gyration

From Rg analysis of all the complexes, it was observed in Fig. 4C that throughout the simulation scale, 2VSM-BA displayed consistent Rg values except for only at 78.2 ns and the average value (21.05 Å) was lower than that of the complexes of 2VSM-Ribavirin (21.06 Å) and 2VSM-MA (21.09 Å).

#### 3.3.4 Solvent-accessible surface area

A receptor's structure and functional characteristics are also influenced by its SASA value [81]. In Fig. 4D, the average SASA value of the 2VSM-Ribavirin was 17428.1 Å^2^ that was less than that of both 2VSM-BA (17492.8 Å^2^) and 2VSM-MA (17684 Å^2^).

#### 3.3.5 Molecular surface area

The available van der Waals contact area expected with a 1.4 probe radius is described by MolSA [82]. In Fig. 4E, the average MolSA value of 2VSM-MA (19384 Å^2^) showed better surface interaction when compared to 2VSM-Rbavirin complex (19055 Å^2^). Despite having low value, the complex 2VSM-BA's average (19035 Å^2^) was nearly similar to the control drug.

#### 3.3.6 Total hydrogen bond number analysis

H-bond analysis showed that both complexes of ligand have a lower number than the 2VSM-Ribavrin (Fig. 4F). The average number of total H-bond for the 2VSM-Ribavrin, 2VSM-BA, and 2VSM-MA were ∼6, ∼2, and ∼5.

### 3.4 Binding free energy results

The free energy differential between the fully unbound and bound states is known as binding free energy [83]. At the beginning of the simulation, the binding energy of 2VSM-Ribavirin, 2VSM-BA, and 2VSM-MA complexes were −258.611, −176.74, and −97.27 kJ/mol (Fig. 4G). After 100 ns, the average binding free energy of 2VSM-MA, 2VSM-BA was −53.13 kJ/mol and −60.08 kJ/mol that was higher than that of the 2VSM-Ribavirin (−180.52 kJ/mol).

### 3.5 Secondary structure analysis

**Fig. 5:**
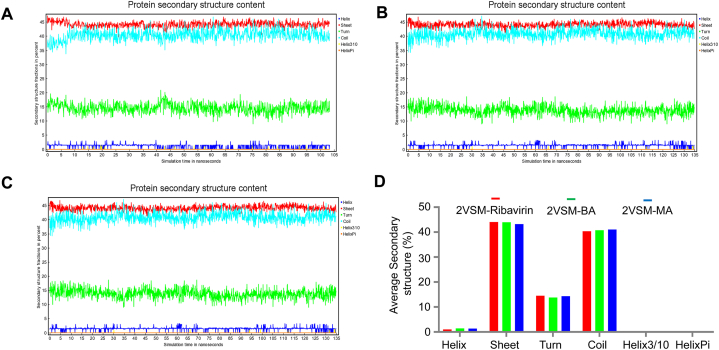
Analysis of Secondary Structures in 2VSM-Ligand Complexes. **A-C**. The 2VSM-Ribavirin, 2VSM-BA, and 2VSM-MA complexes' secondary structural components_helices, beta sheets, and turns_remain stable during the course of the 100 ns simulation, suggesting that the protein integrity was maintained. Note: Graph HelixPi has all zero values. **D**. These elements' quantification revealed about the same percentages in every compound, indicating that ligand interaction did not cause any notable conformational changes. As evidence of their persistent connections and potential as powerful inhibitors with long-lasting inhibitory effects, the ligands continuously stayed attached to the inhibition site.

To thoroughly investigate the conformational changes induced by ligand binding, we performed a detailed secondary structure analysis of three distinct complexes. Our findings, illustrated in Fig. 5(A–C), indicate that the key secondary structural elements—specifically helices, beta sheets, and turns—remained highly consistent throughout the entire simulation period. This stability suggests that both the control complex (2VSM-Ribavirin) and the ligand-bound complexes (BA and MA) exhibit robust structural integrity.

In Fig. 5D, we quantified the secondary structure elements across all three complexes, revealing that the percentages of helices, beta sheets, and turns are nearly identical. This similarity reinforces the idea that the overall architecture of the protein is preserved, despite the presence of different ligands.

Furthermore, our observations show that the ligands consistently remain attached to the inhibition site throughout the simulations, without any significant structural modifications. This consistent binding behavior implies a high degree of stability for the ligands within the active site, suggesting that they effectively maintain their conformations while exerting their inhibitory effects. Collectively, these results highlight not only the reliability of the structural data but also the potential effectiveness of the ligands in modulating protein function through stable interactions.

## 4 Discussion

Nipah virus may emerge as a pandemic in the future, as almost 16 outbreaks have been reported till now. The outbreaks resulted in 40–70 % of death, with some outbreaks exhibiting a 100 % case fatality rate [84]. The high fatality rate urges to search for novel anti-Nipah drugs that paved the way to work with the viral protein as a potential target for antiviral therapy. Therefore, cell surface-associated NiV-G that aids the entry of the virus into host cells by attaching to the human ephrin-B2 receptor was chosen as a target [85,86].

Since propolis has a diversified mixture of compound with wide range of therapeutic efficacy including antiviral activity against HSV-1, acyclovir-resistant HSV1, HSV-2, adenovirus type 2, vesicular stomatitis virus, and polio virus-2 in vitro and/or animal models [87], in this study, we performed computational studies to evaluate the binding efficacy of antiviral compounds of propolis towards the NiV-G protein and enact MD simulations to get deeper understanding in terms of their structure and the microscopic interactions. The safety profile of the antiviral compounds of propolis were established by pharmacokinetic and toxicity analysis. Molecular docking showed BA and MA bound more effectively than the control, ribavirin. Notably, flavonoids in propolis, such as quercetin, fisetin, luteolin, and Plukenetione A, which are known for their antiviral and anti-inflammatory properties, exhibited significant binding affinities with docking scores above 8. These compounds may complement the effects of BA and MA by reducing inflammation and enhancing viral suppression, thereby presenting a multifaceted approach to therapeutic development [[88], [89], [90]]. To validate our findings, we compared the binding properties of our experimental molecules with already reported anti-Nipah inhibitors including remdesivir, favipiravir, and acyclovir. We observed that remdesivir binds to NiV-G with a binding affinity of 7.5 kcal/mol [91], favipiravir with 3.70 kcal/mol [92], and acyclovir with 6.55 kcal/mol [93]. These results indicate that both propolis-derived compounds, BA and MA, exhibit higher binding affinities toward the NiV-G protein compared to the established inhibitors.

To further verify the protein-ligand non-bond interaction, MD simulation - creates an environment corresponding to human physiological environment - was executed for 100 ns. The average value of the MD simulation data is summarized in Table 4. In MD studies, both 2VSM-BA and 2VSM-MA revealed less RMSD value compared to 2VSM-Ribavirin, suggesting that these complexes have more stable structures during the 100 ns simulation [94]. In terms of residual instability, the intensity and average values were high in case of 2VSM-Ribavirin. RMSF data confirmed that local residual fluctuation intensity was more stable in the 2VSM-BA and 2VSM-MA [95]. The Rg values highlighted that 2VSM-BA had greater compactness and tightness compared to the 2VSM-Ribavirin and 2VSM-MA, representing less gyration and spin when bound to BA [96]. Though the average SASA of 2VSM-MA was a little bit higher, the mean value of 2VSM-BA was almost similar to that of the 2VSM-Ribavirin. A lower SASA value implies better accessibility for both ligands (BA and MA) to interact with the NiV-G protein [97]. Average molecular Surface Area (MolSA) value of 2VSM-BA was slightly lower than that of the 2VSM-Ribavirin, designating a reduction in the interacting surface area, while 2VSM-MA exhibited an increase, signifying a potential influence on ligand binding [98]. 2VSM-BA and 2VSM-MA exposed less binding strength as they formed diminished number of hydrogen bonds compared to 2VSM-Ribavirin [99]. However, the hydrogen bonds of 2VSM-BA exhibited greater stability compared to that of 2VSM-MA (Fig. 4F). Besides, the latter two compounds showed relatively similar stability profile.

**Table 4:** Summary of average score of MD simulation parameters.

Protein-ligand	2VSM-Ribavirin	2VSM-BA	2VSM-MA
RMSD (Å)	1.424	**1.138 ↓**	**1.297↓**
RMSF (Å)	1.076	**1.035 ↓**	**1.023↓**
Rg (Å)	21.061	**21.058 ↓**	21.095 ↑
SASA (Å^2^)	17428.13	**17492.77 σ**	17684.73 **↑**
MolSA (Å^2^)	19054.8	**19035.3 σ**	**19384 ↑**
Total H-Bonds	∼6	∼2 ↓	**∼5** ↓
MM-PBSA (kJ/mol)	−180.520	**−60.082 ↑**	**−53.133 ↑**

Here, ‘↑’ = higher than 2VSM-Ribavirin; ‘↓’ = lower than 2VSM-Ribavirin; ‘σ’ = almost same as 2VSM-Ribavirin.

Overall, all the binding poses measured in MD simulation proved favorable binding interaction between experimental ligands and NiV-G protein. Since the pentacyclic compounds bind with the biomolecules strongly than the tetracyclic and tricyclic ones, the pentacyclic structures of BA and MA may contribute to the better MD results [100].

MM/PBSA approach, which computes the molecular mechanics energies in association with the continuum solvation free energy and conformational entropy of a ligand-biomolecule complex, is central to the MD simulation as it processes conformational trajectories produced over different time sets during simulation and by this way, it transcribes and rationalizes the empirical findings of the virtual screening [101,102]. MM-PBSA (Molecular Mechanics Poisson-Boltzmann Surface Area) calculation of both 2VSM-BA and 2VSM-MA exhibited relatively higher values, indicating potentially stronger binding affinities compared to 2VSM-Ribavirin.

Our study investigated the conformational changes induced by ligand binding in three protein complexes, showing that key secondary structural elements—helices, beta sheets, and turns—remained stable throughout the simulation. This consistency indicates that both the control complex (2VSM-Ribavirin) and the ligand-bound complexes (BA and MA) maintain their structural integrity and functionality. The nearly identical percentages of these elements across the complexes reinforce the robustness of the protein architecture. Additionally, the ligands demonstrated stable binding to the inhibition site without significant structural changes, suggesting favorable interactions that enhance their affinity for the target protein. Overall, these findings highlight the potential of these ligands as effective inhibitors, laying the groundwork for future research on their dynamics and implications for drug development.

Our current findings provide a valuable insight into the dynamic behavior and interactions of the experimental protein-ligand complexes. To support our study results, we compared the molecular interaction profiles of our top hit compounds with the already testified molecules on different NiV proteins. Surprisingly, propolis-derived BA and MA showed better molecular interaction than those in the published literatures (Table 5) [43,[103], [104], [105]]. Previously reported in vitro and in vivo antiviral activity of BA and MA also corroborates our in silico results. Besides, BA and MA are classified as triterpenoids, which are reported to impede viral entry into host cells by preventing early-stage adsorption and invasion, consequently inhibiting post-infection viral replication [106]. Earlier research exhibited that BA has significant anti-pseudorabies virus (SuHV-1, suid herpes virus) activity in vitro [63]. MA demonstrated in vitro anti-HIV activity in H9 lymphocytes [53,107], in vitro and in vivo anti-HSV activity [108], and also anti-Epstein–Barr virus activity [54]. Therefore, this study predicts the binding ability of BA and MA with the NiV-G protein offering a basis for further investigation and optimization of drug design strategies.

**Table 5:** Comparison of the docking scores of propolis-derived hit molecules (BA and MA) against various NiV proteins with those of molecules reported in previous studies.

Protein & PDB ID	Reported top hit molecules with docking scores (kcal/mol)	BA (kcal/mol)	MA (kcal/mol)
NiV-F (1WP7)	4-hyroxypanduratin A = 4.83 [103]	6.91	6.08
NiV-N (4CO6)	RSV604 = 9.00 [43]	9.34	8.21
NiV-F (5EVM)	RSV604 = 8.60 [43]	8.99	8.21
NiV-G (2VSM)	RSV604 = 8.80 [43]	9.84	9.58
NiV-P (6EB9)	Neoandrographolide = 6.00 [104]	7.48	6.71
NiV-G (2VWD)	Neoandrographolide = 8.10 [104]	8.54	8.21
NiV-M (7SKT)	Tribulusamide B = 8.66 [105]	8.51	8.08

YASARA-generated higher positive score suggests the least binding free energy.

However, computational methods have inherent limitations that should be considered. Molecular docking, molecular dynamics (MD) simulations, and MM-PBSA calculations operate in controlled environments that fail to replicate the complexity of biological systems, including cellular processes, systemic interactions, and host-pathogen dynamics. Additionally, computational constraints, such as limited simulation times and the accuracy of force fields, further reduce the reliability of these approaches [109,110]. To address these limitations, future studies should include experimental validation of the predicted binding affinities through in vitro and in vivo antiviral assays. These complementary validation strategies aim to strengthen the translational relevance of the present findings and advance the development of effective therapies against NiV.

## 5 Conclusion

The present study highlights the promising potential of small molecules in propolis as antiviral agents against the Nipah virus, specifically targeting the host cell attachment protein (NiV-G). Molecular interaction analysis revealed strong binding affinity between the compounds, MA and BA, and the target protein, supported by stable complexes observed in 100 ns MD simulations and robust binding affinity estimated through MM-PBSA calculations. Given the established antiviral activity of MA and BA, our findings underscore the importance of further in vivo and in vitro studies to explore their efficacy as anti-NiV inhibitors. This conclusion may emphasize a significant step forward in the development of potential therapeutics against Nipah virus infection.

## CRediT authorship contribution statement

**Muaz Faruque:** Writing – original draft, Methodology, Investigation, Data curation. **Md Afjalus Siraj:** Writing – review & editing, Validation, Formal analysis. **Md Nazmul Hasan Zilani:** Writing – review & editing, Methodology, Conceptualization. **Asish Kumar Das:** Writing – review & editing, Methodology, Formal analysis, Conceptualization. **Md Anisuzzman:** Writing – review & editing, Validation, Formal analysis. **Md Monirul Islam:** Writing – review & editing, Validation, Supervision, Software, Resources, Formal analysis, Conceptualization.

## Data availability

The data presented in this study are available in the article.

## Funding

The research work was partly supported by the 10.13039/100020628Information and Communication Technology (ICT) Division, Government of the People's Republic of Bangladesh (Grant ID: 56.00.0000.053.20.019.21–134).

## Declaration of competing interest

The authors declare that they have no known competing financial interests or personal relationships that could have appeared to influence the work reported in this paper.
