# Supplementary material for: Investigating small molecules in propolis as Nipah virus glycoprotein (NiV-G) inhibitors through molecular interaction studies
Source: Heliyon. 2025 Feb 10;11(4):e42595. doi: 10.1016/j.heliyon.2025.e42595 (PMC11883394; doi:10.1016/j.heliyon.2025.e42595)
Supplement: Multimedia component 1 [file mmc1.pdf]

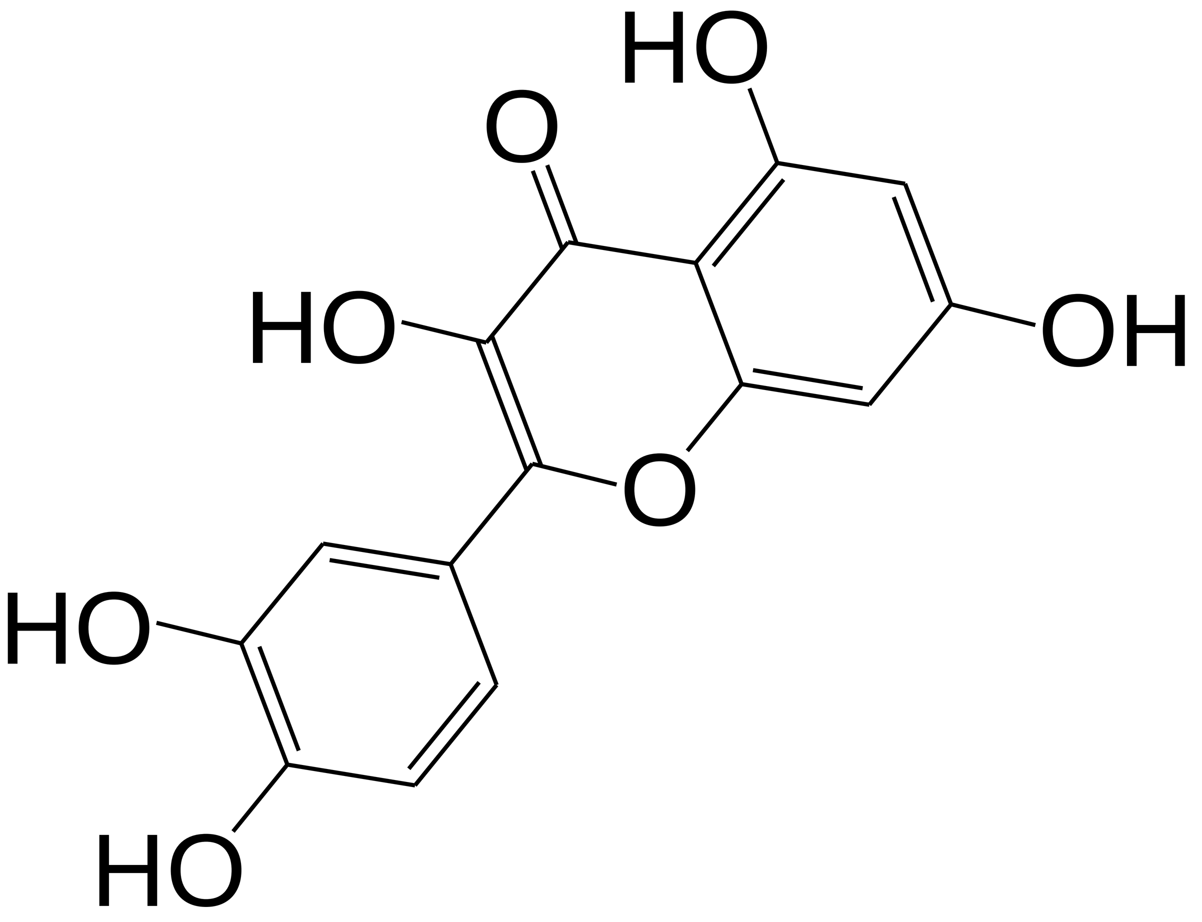

Quercetin

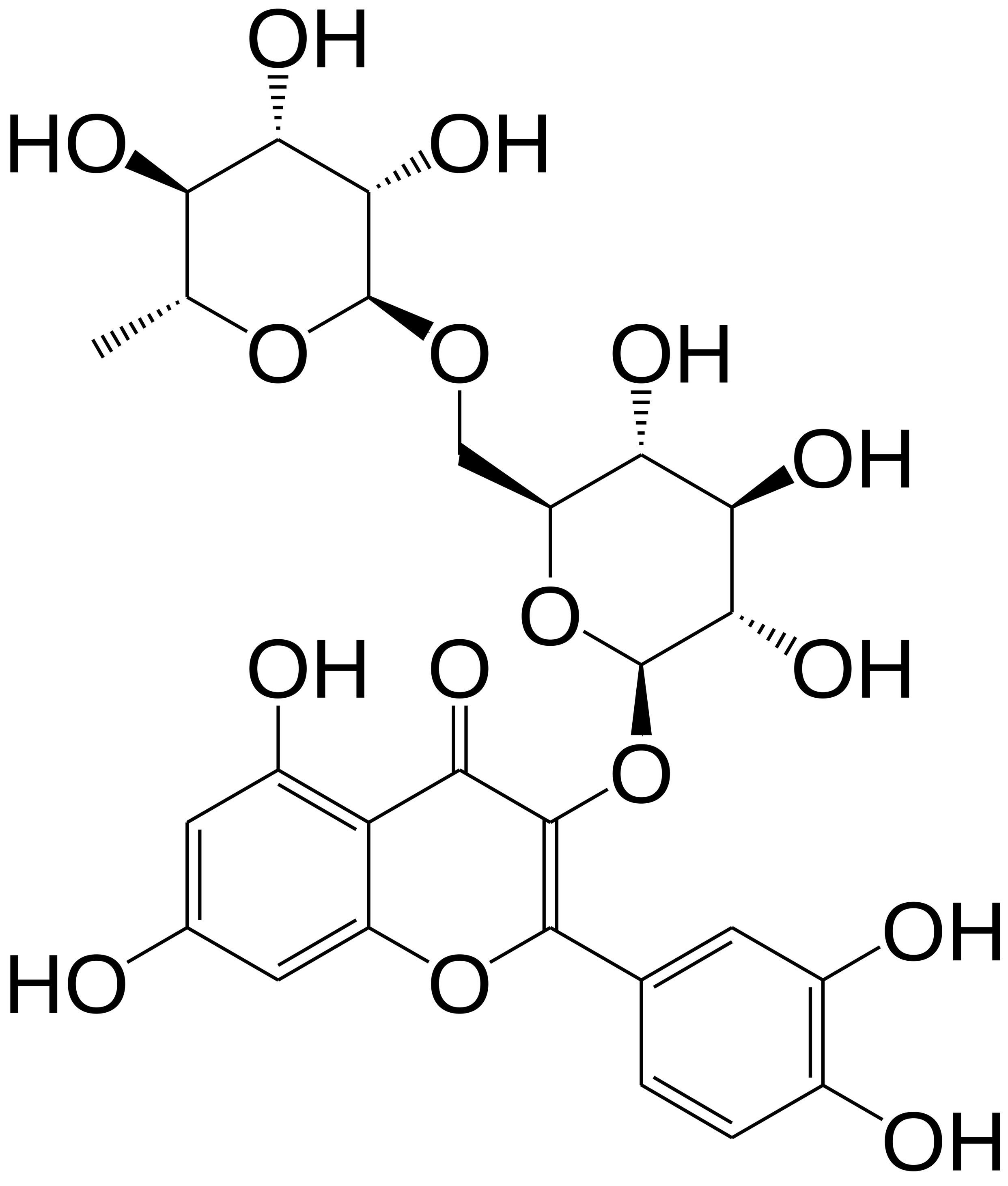

Rutin

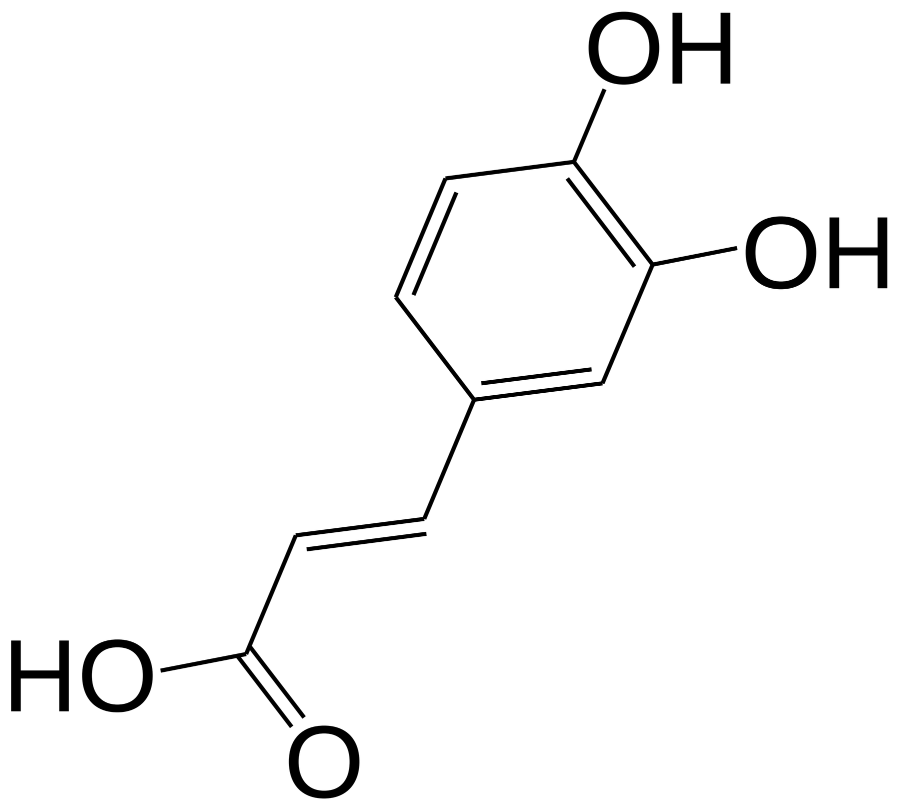

Caffeic acid

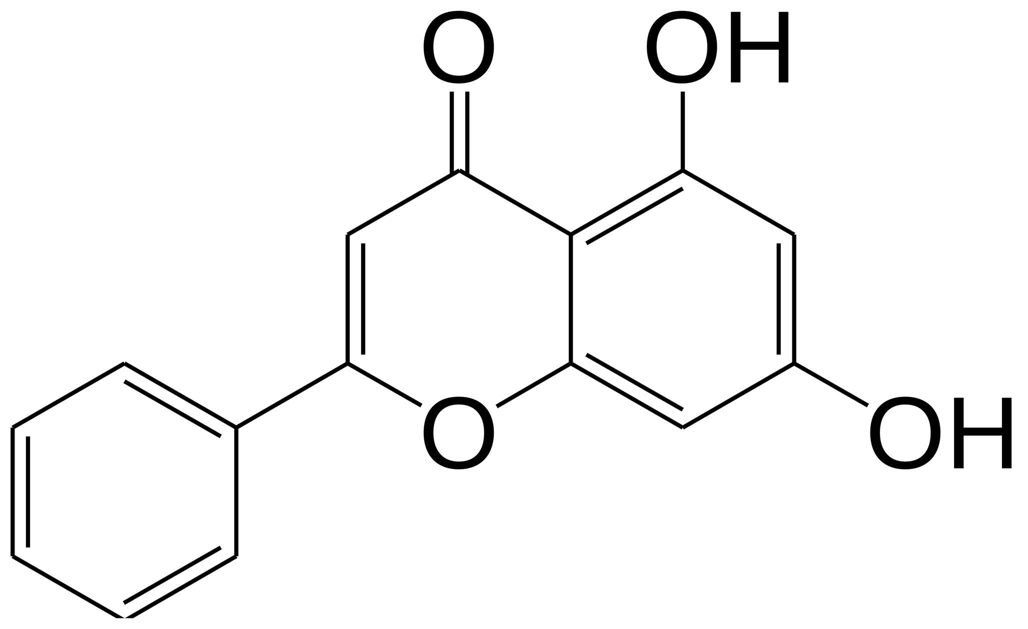

Chrysin

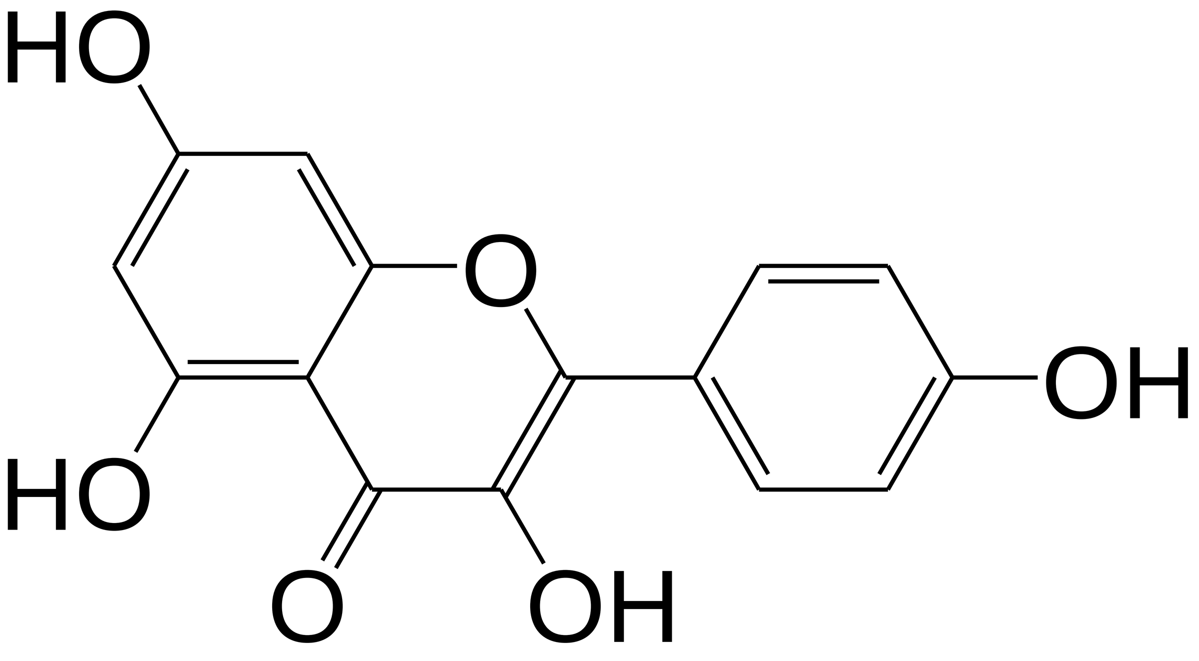

Kaempferol

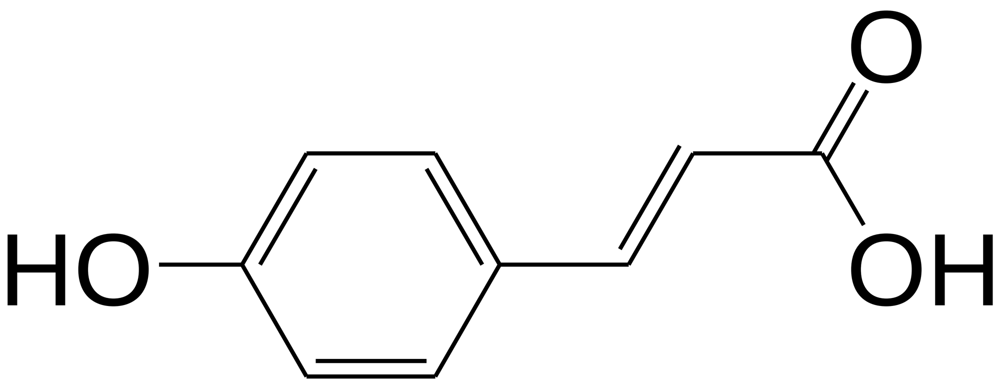

p-Coumaric acid

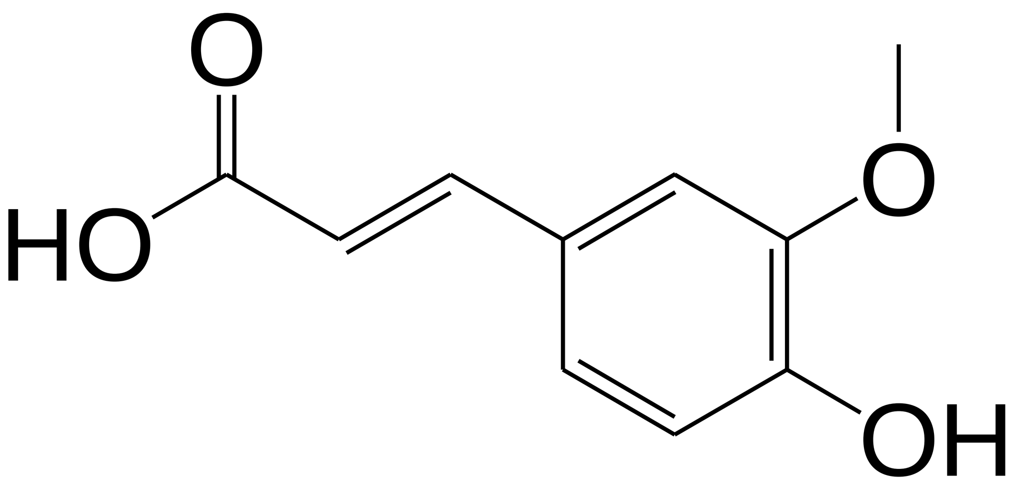

Ferulic acid

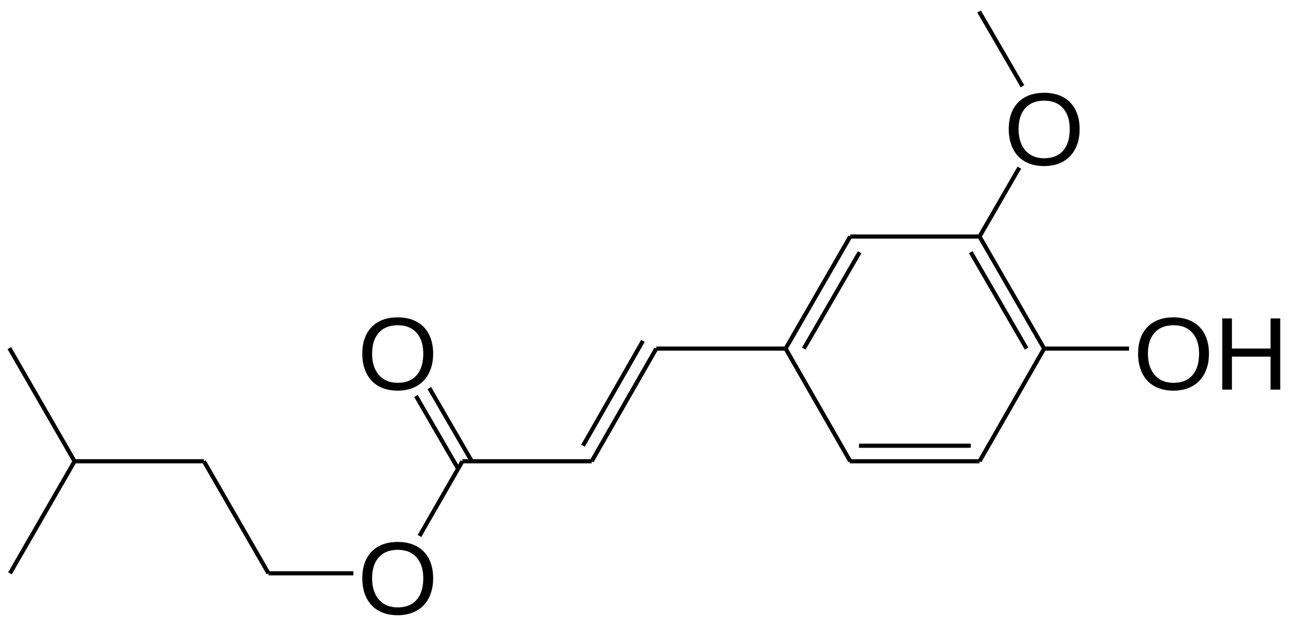

Isopentyl ferulate

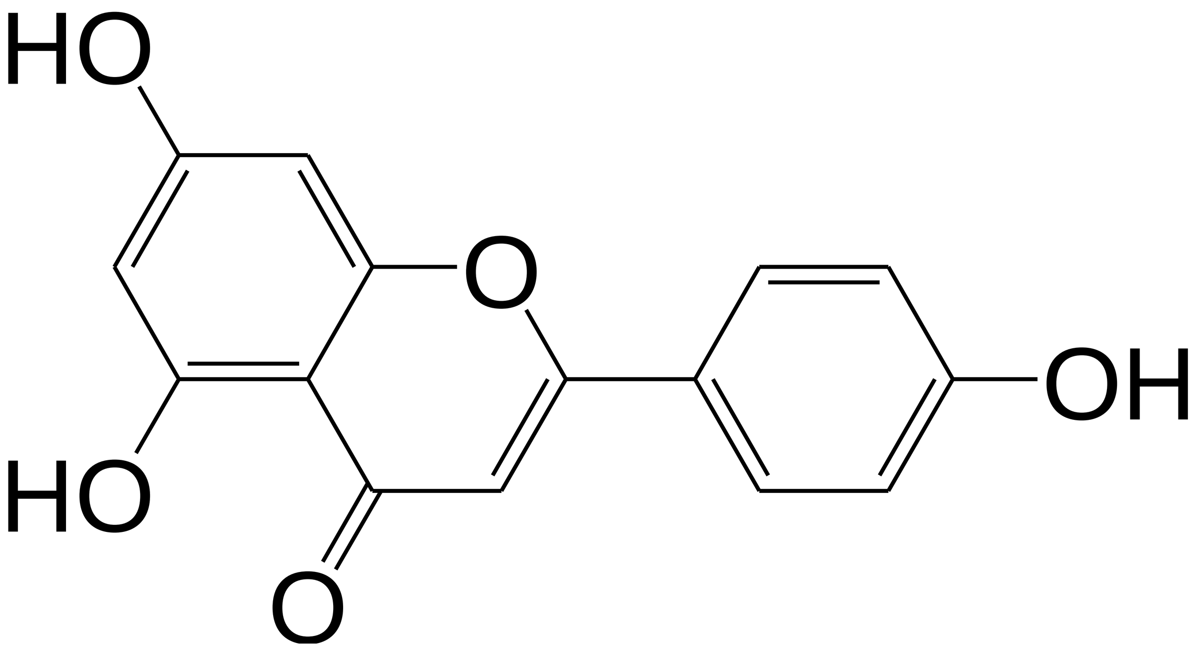

Apigenin

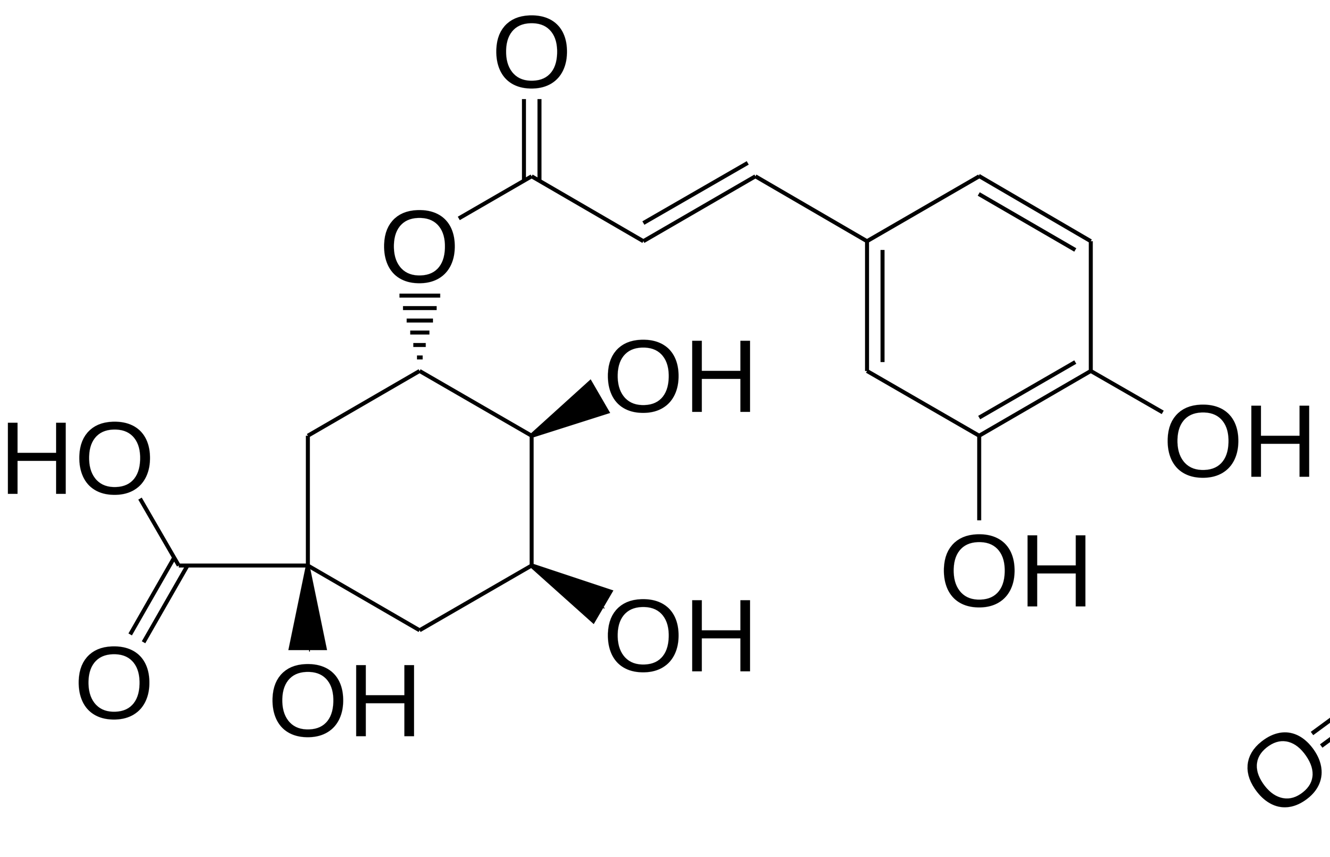

Chlorogenic acid

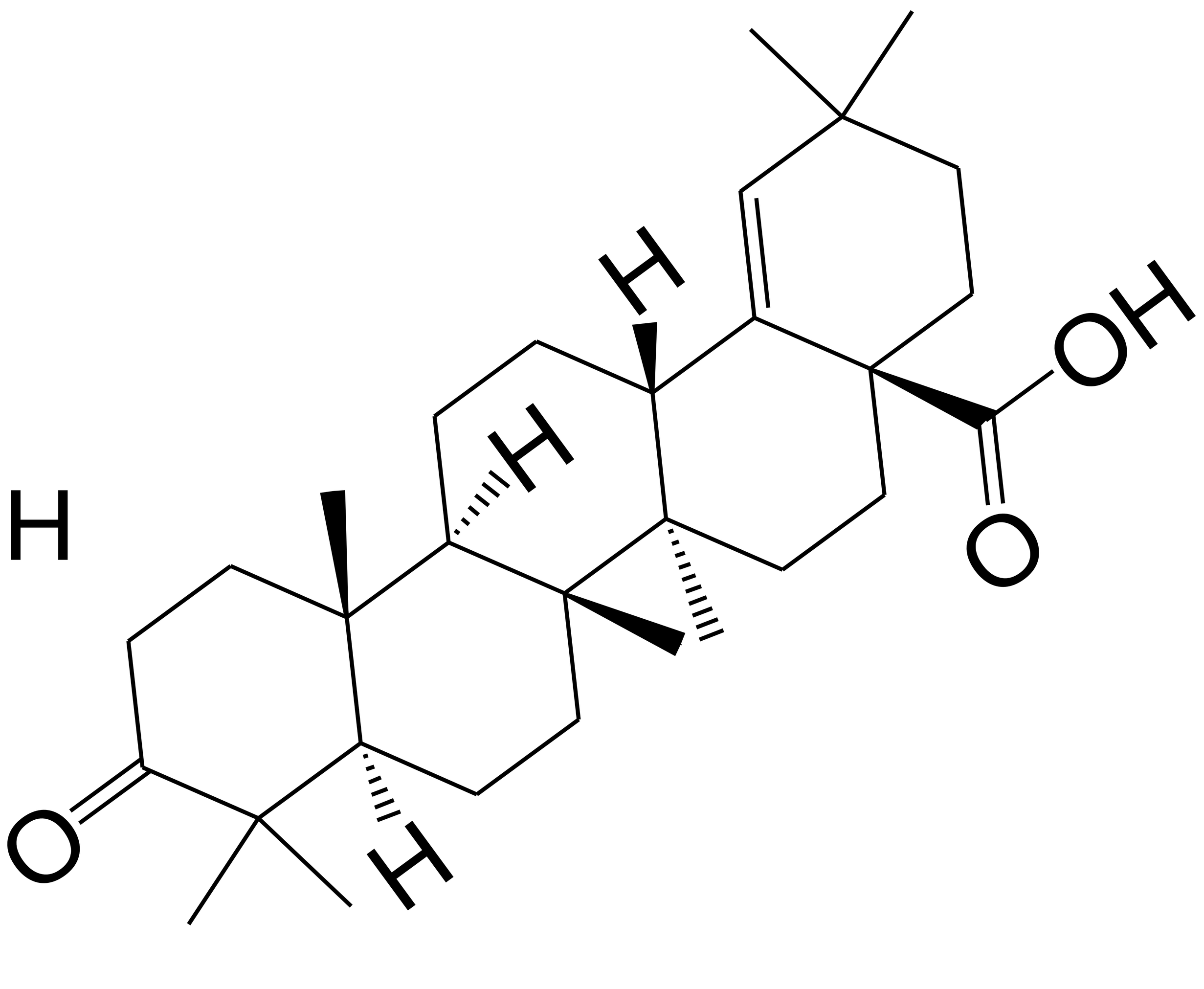

Moronic acid

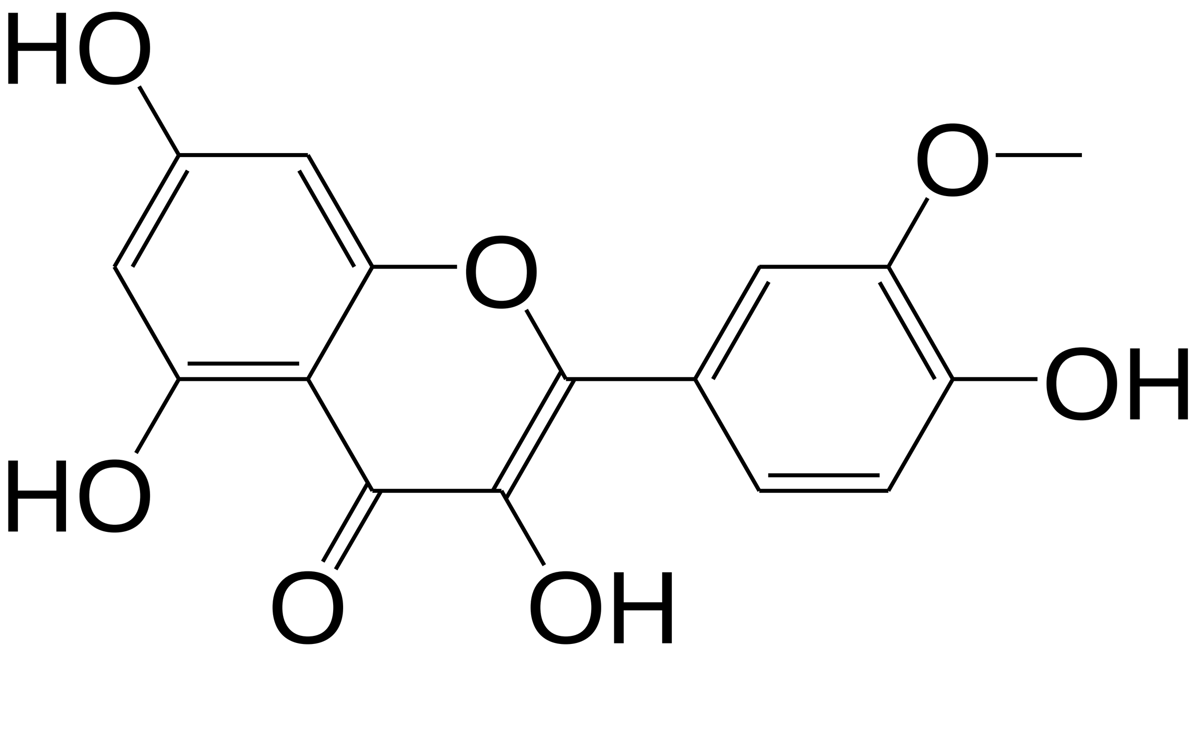

Isorhamnetin

Supplementary Figure 1: Antiviral compounds of propolis

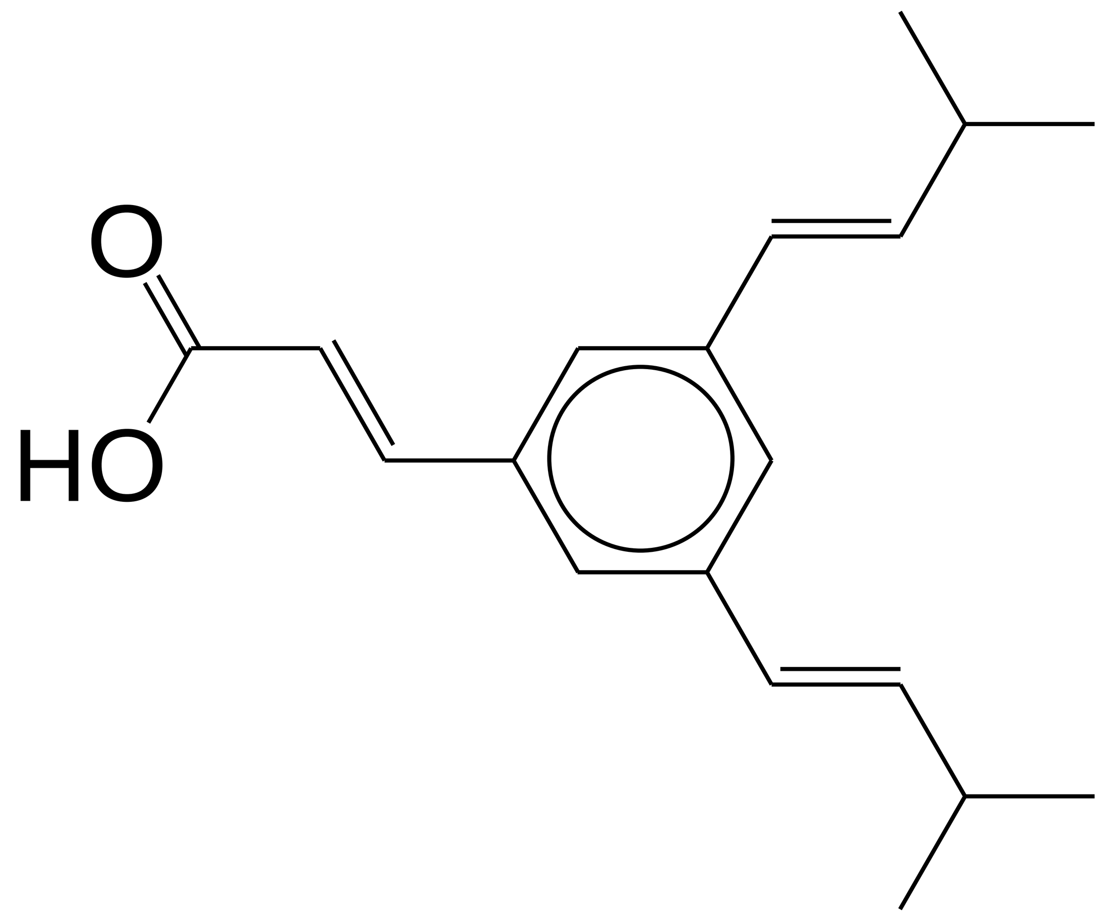

Diprenylcinnamic acid

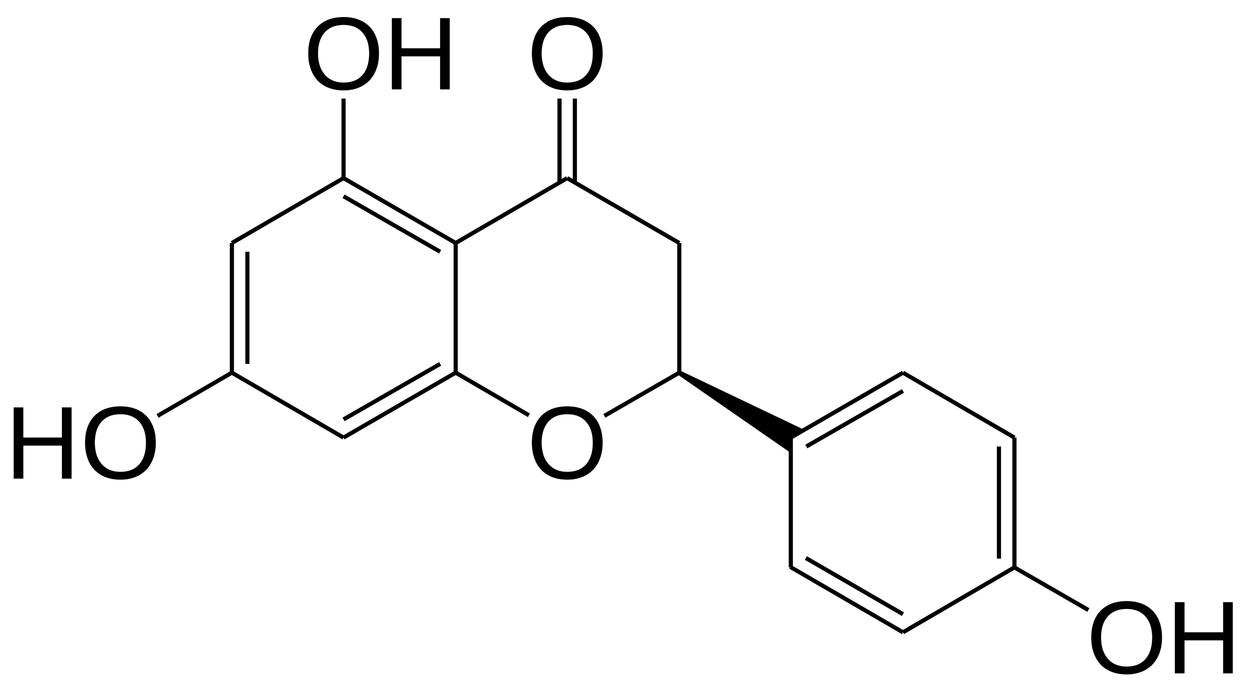

Naringenin

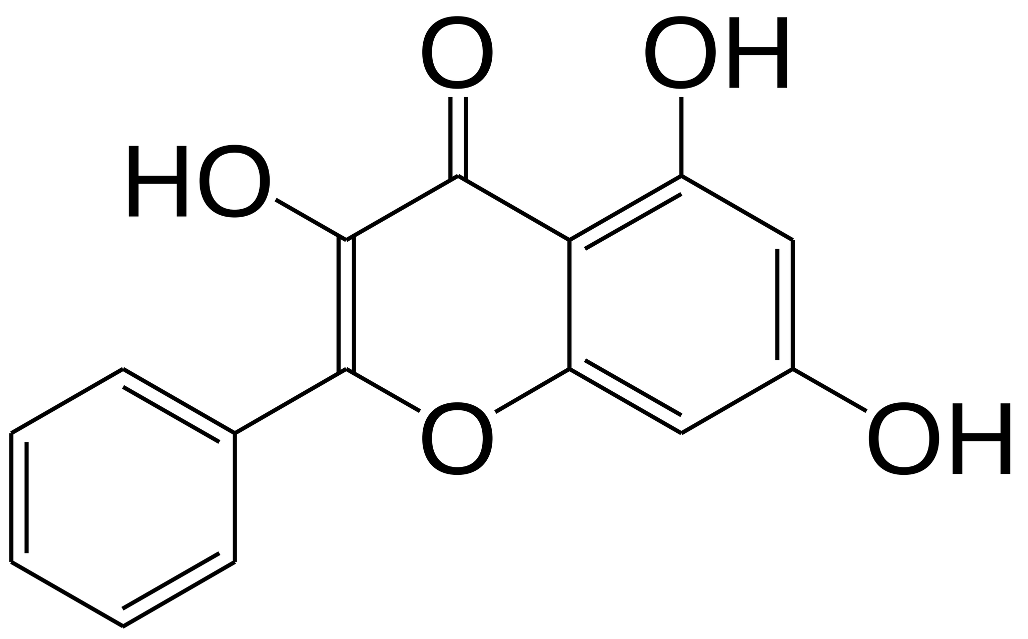

Galangin

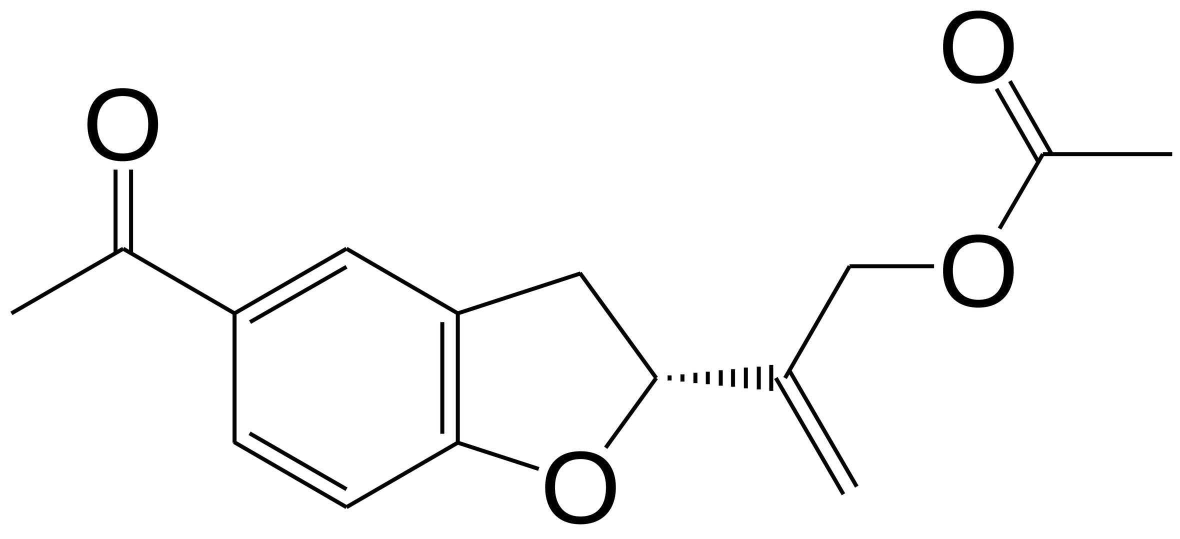

12-Acetoxytremetone

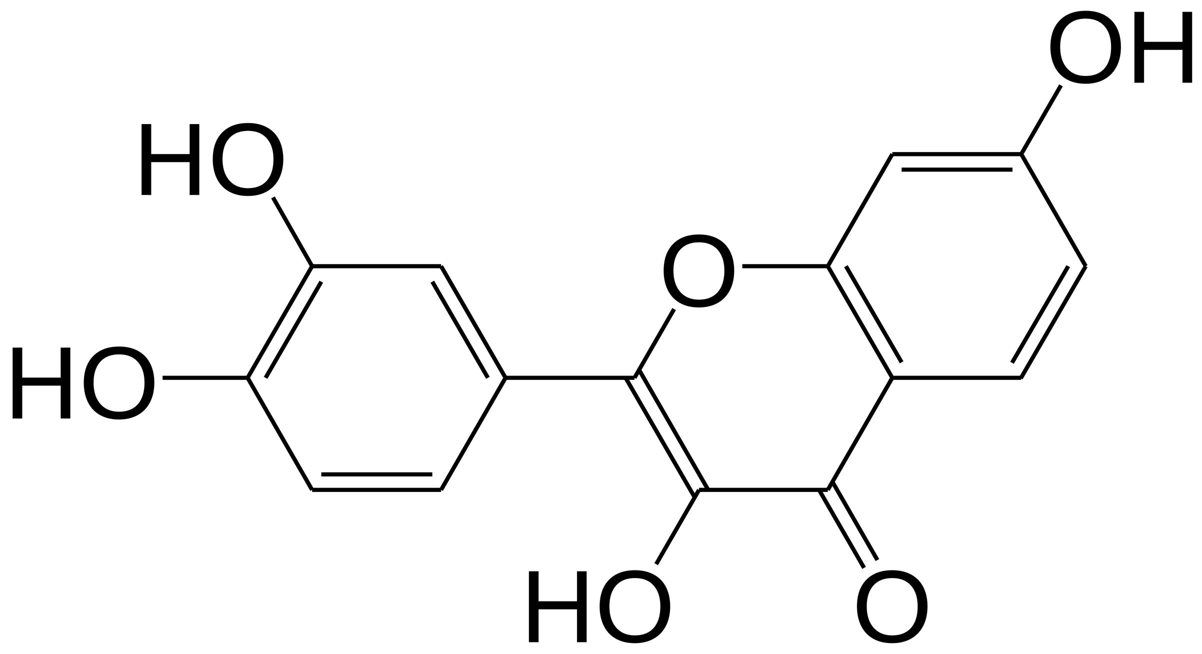

Fisetin

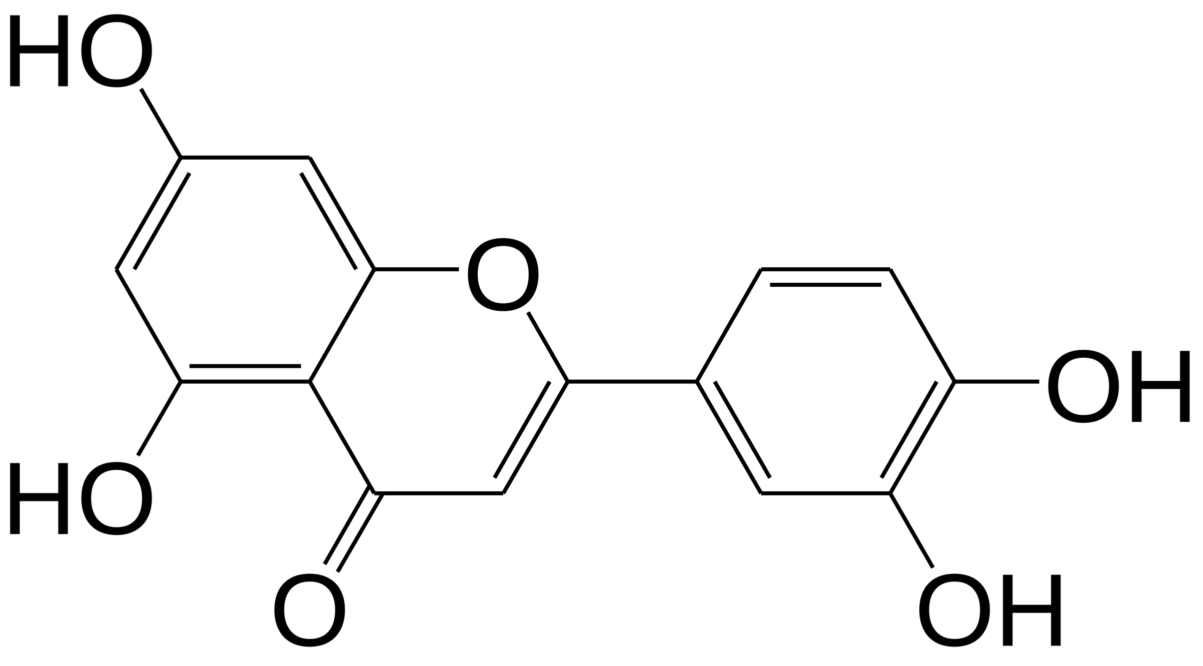

Luteolin

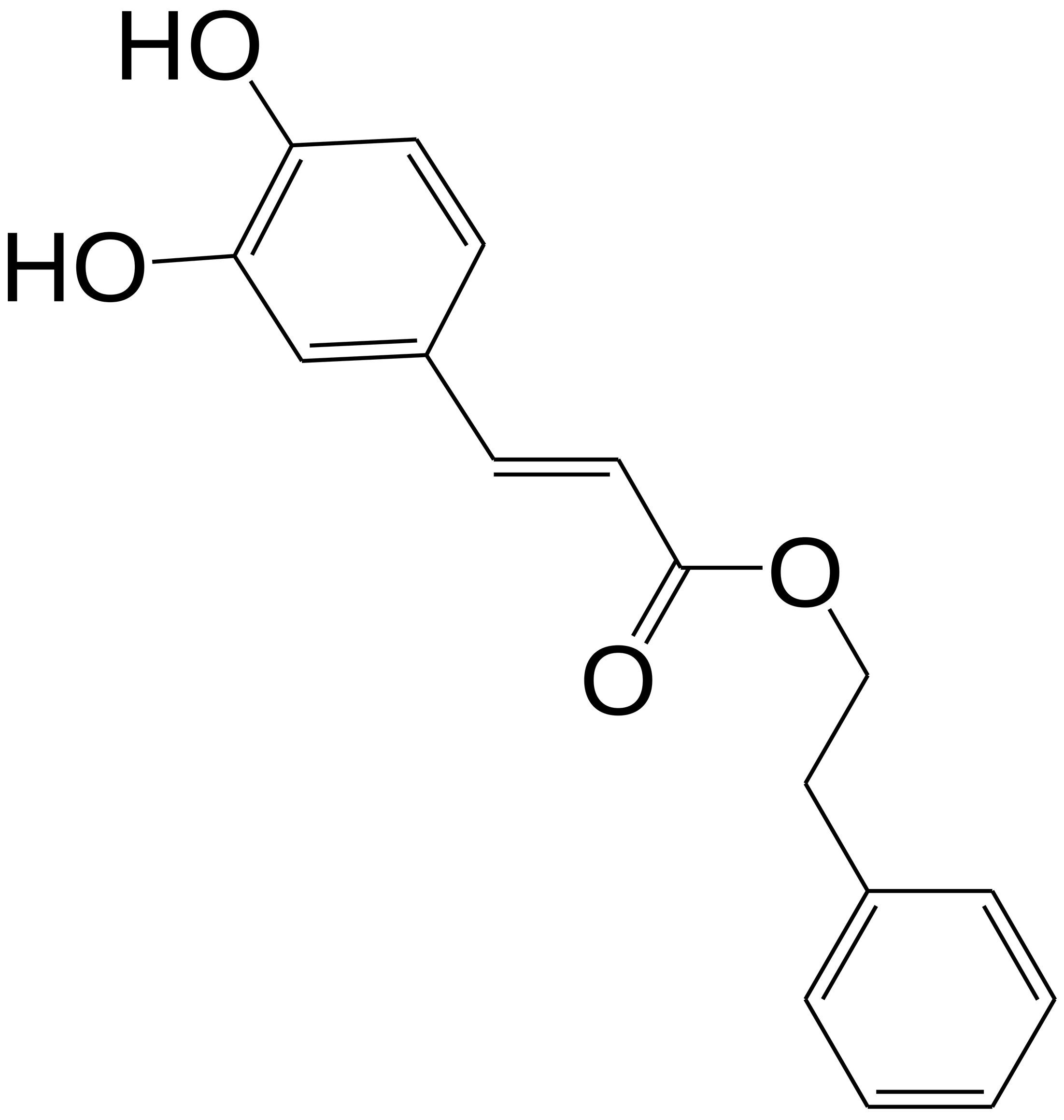

Caffeic acid phenethyl ester

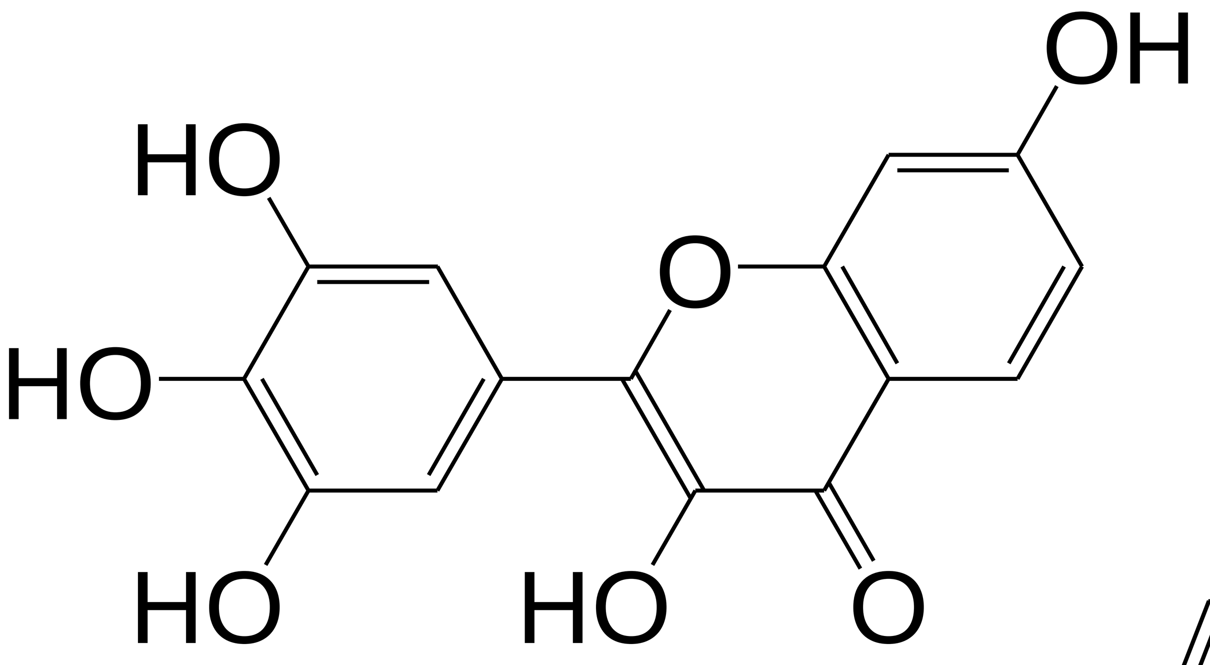

Robinetin

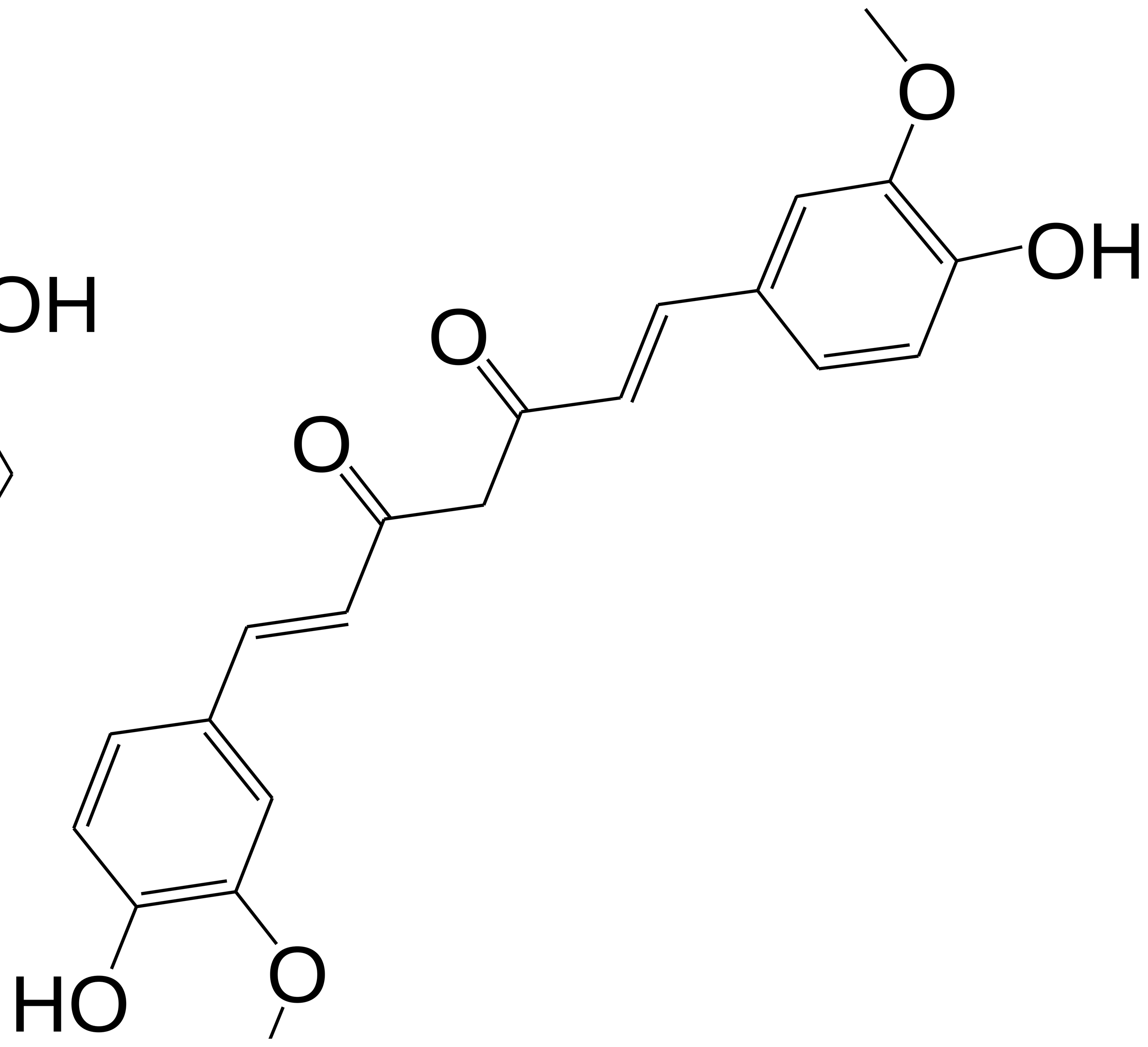

Curcumin

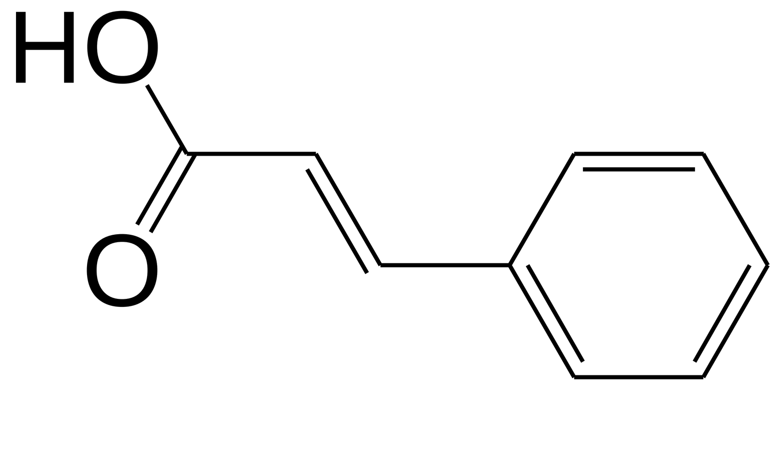

Cinnamic acid

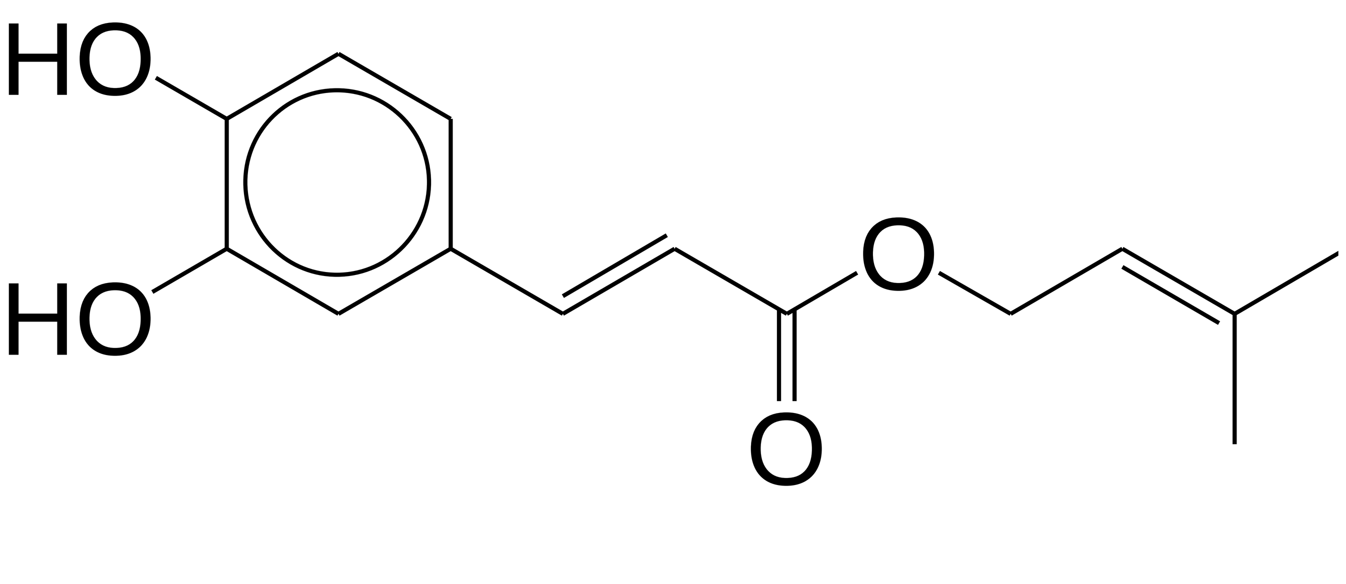

3-methyl-but-2-enyl caffeate

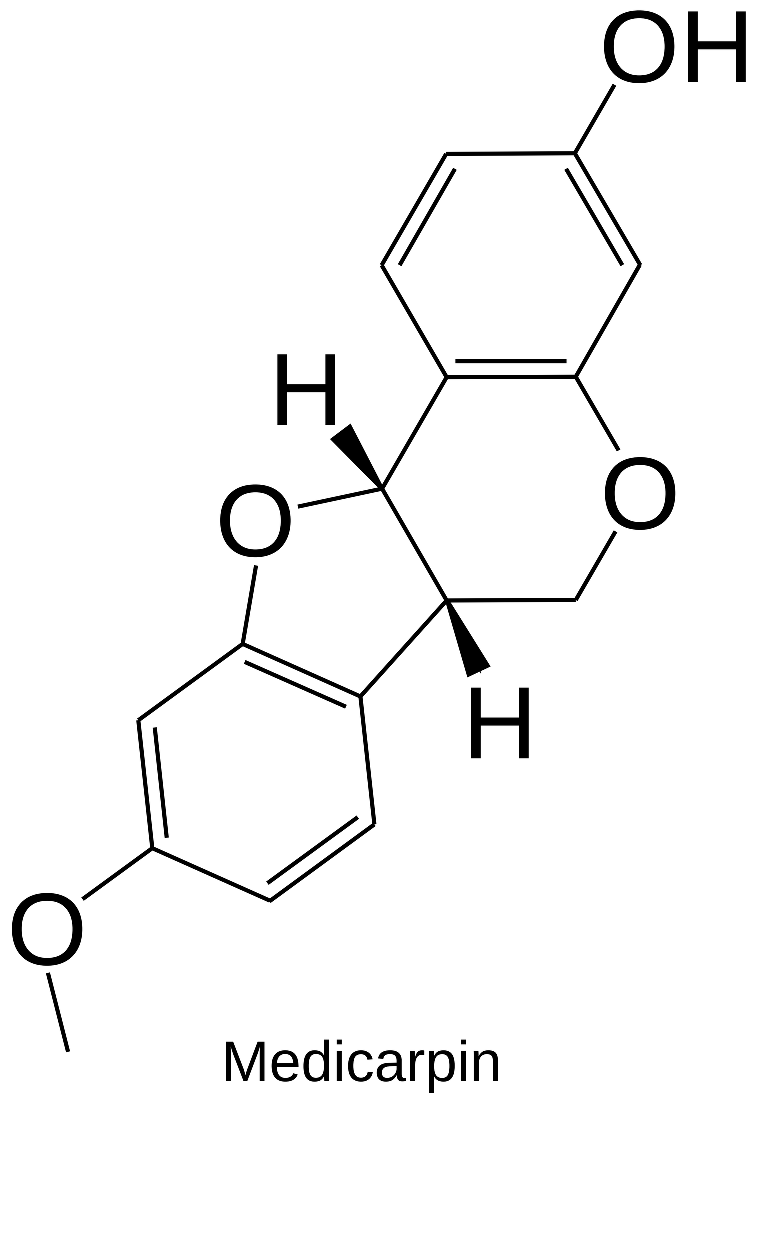

Medicarpin

Supplementary Figure 1: Antiviral compounds of propolis (continued)

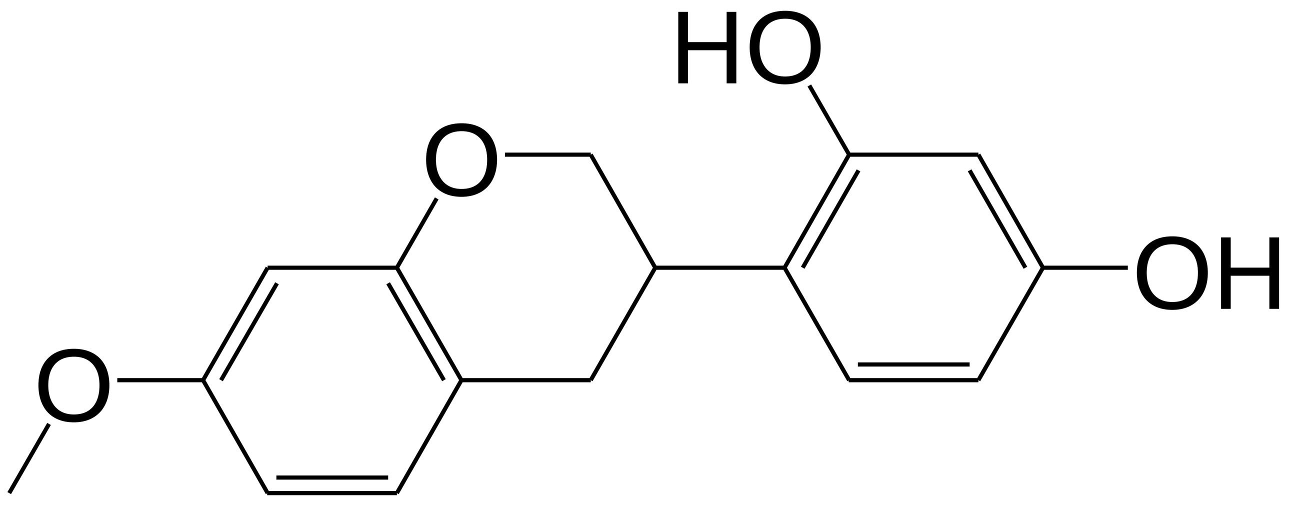

Neovestitol

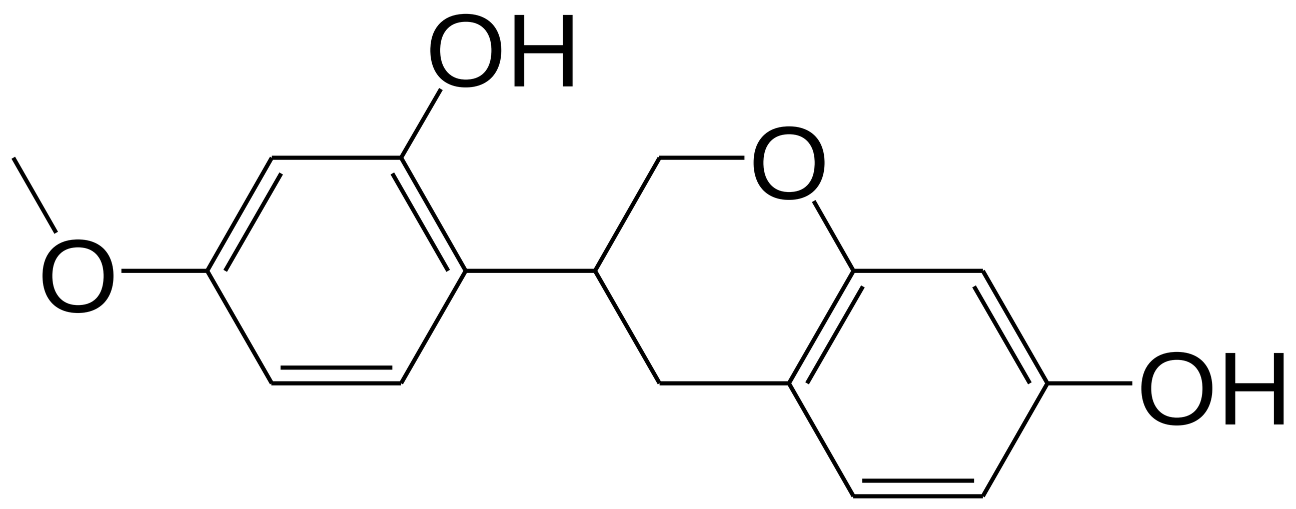

Vestitol

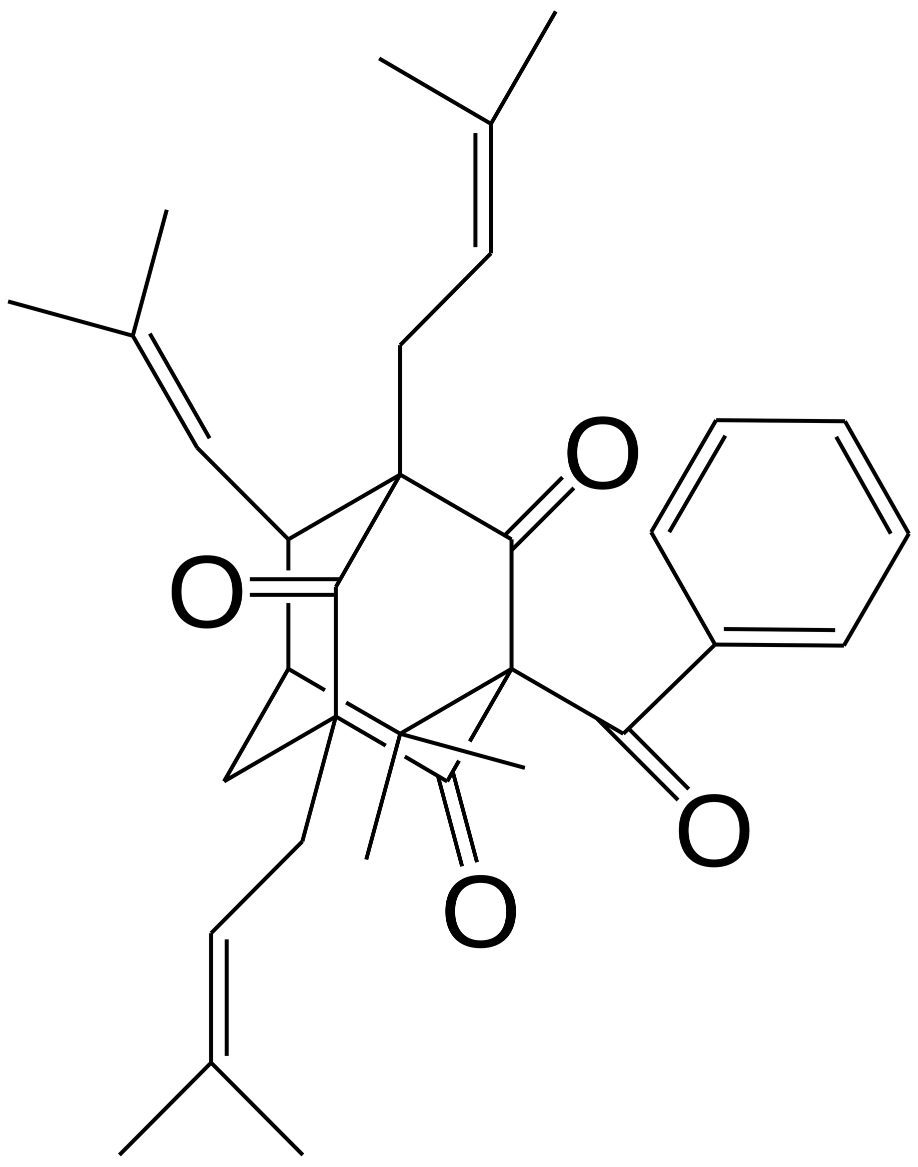

Plukenetione A

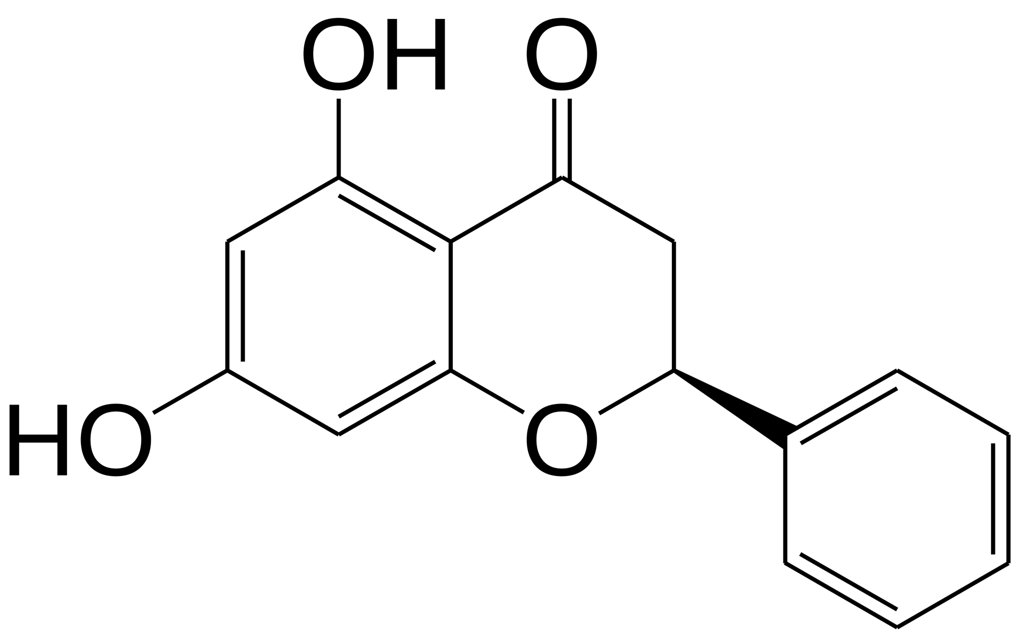

Pinocembrin

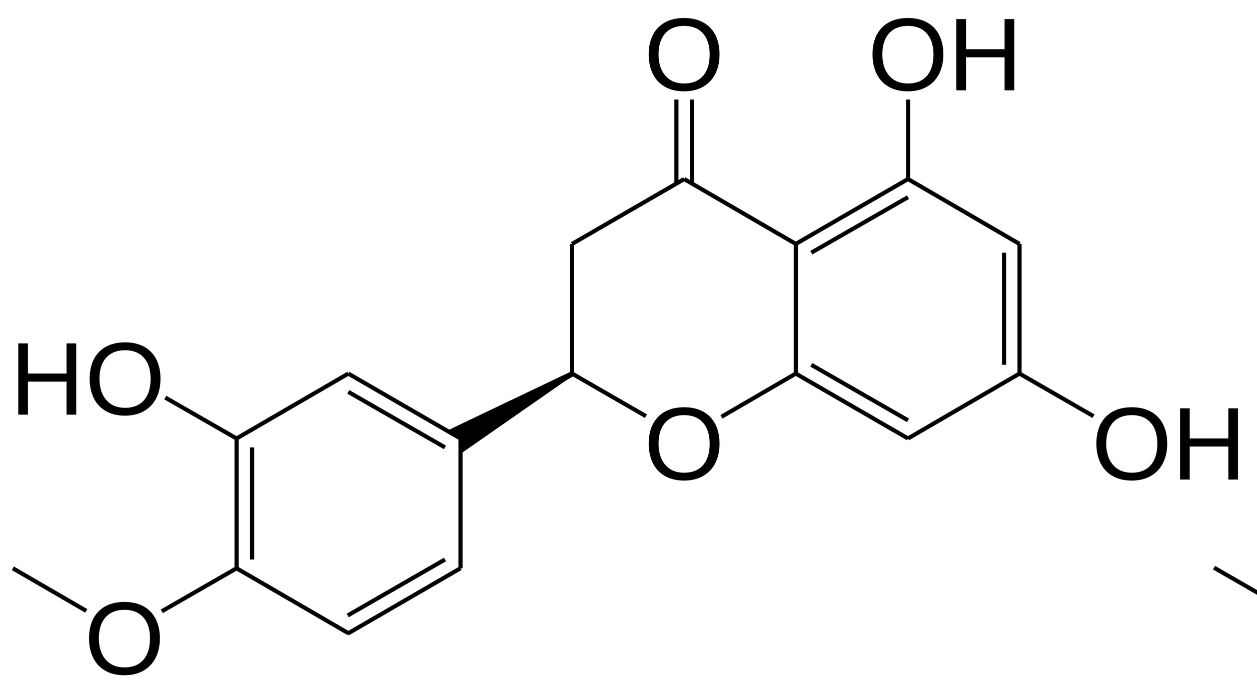

Hesperetin

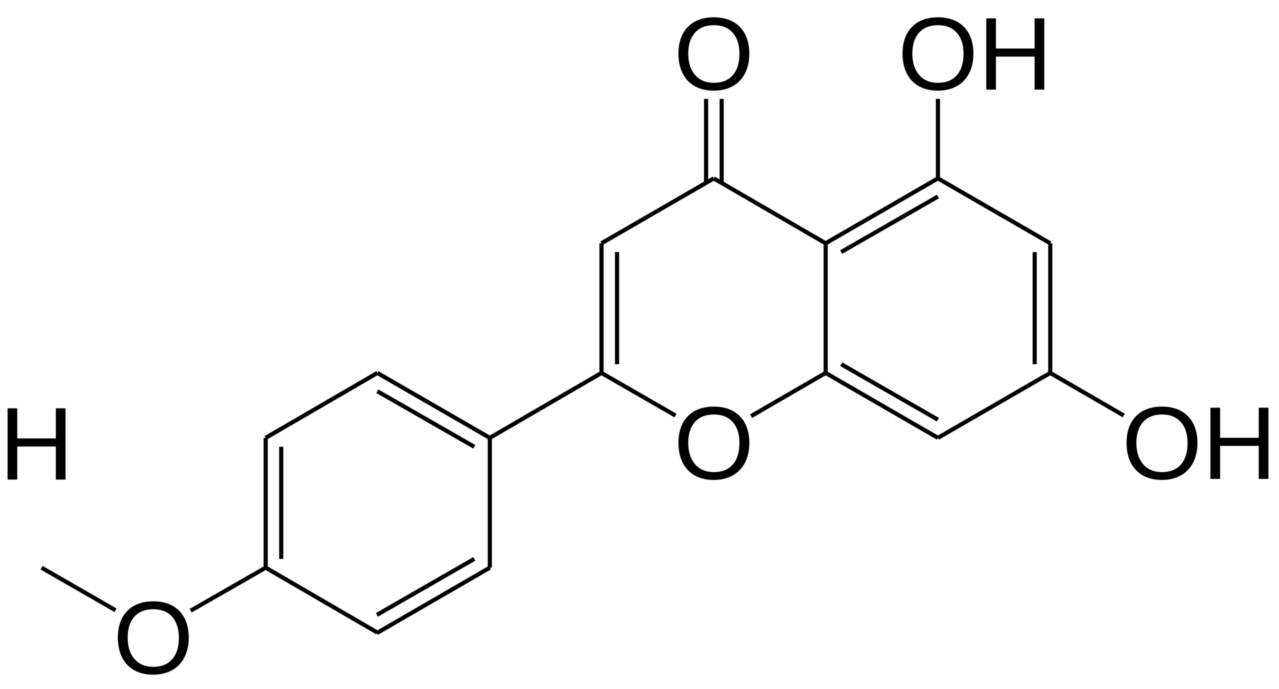

Acacetin

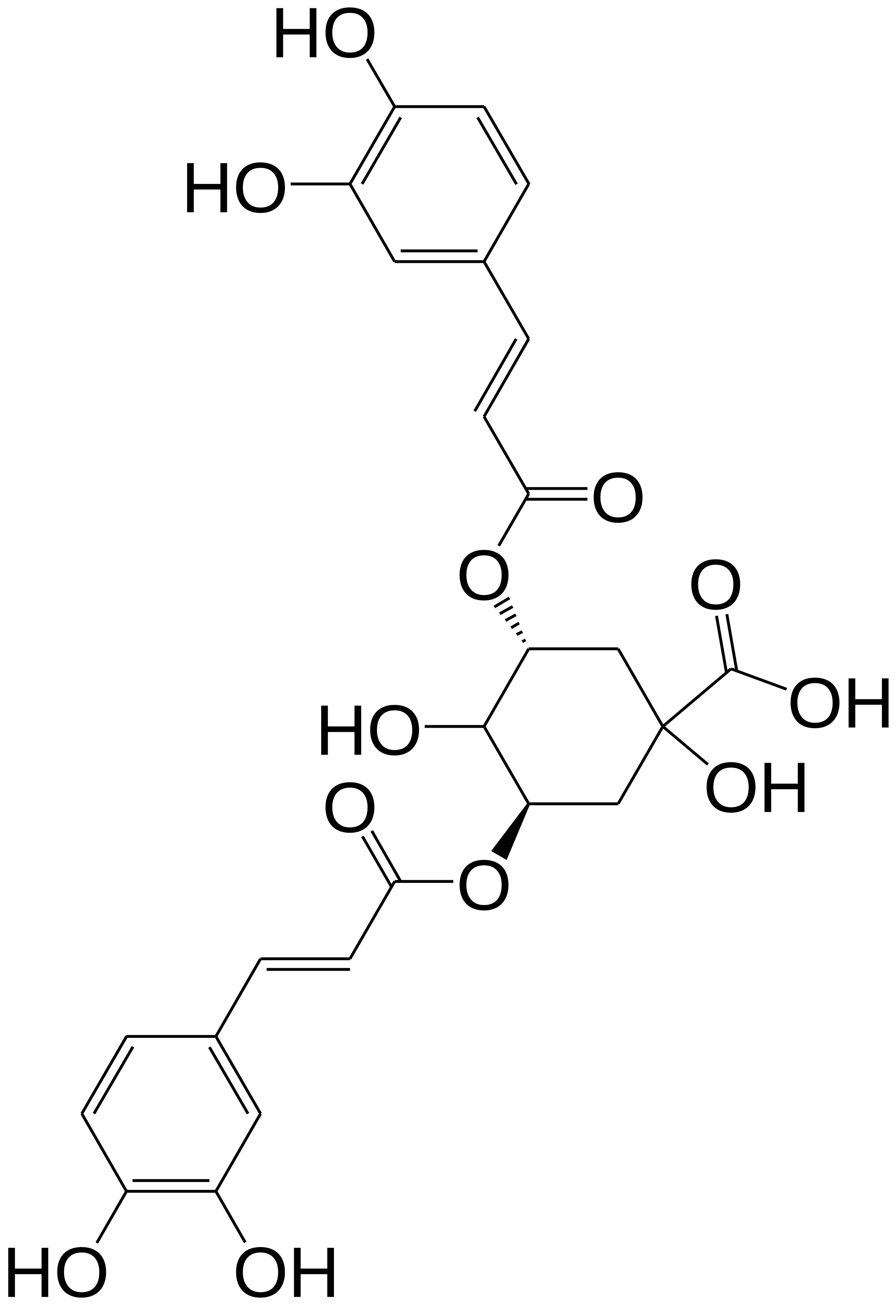

Pinostrobin

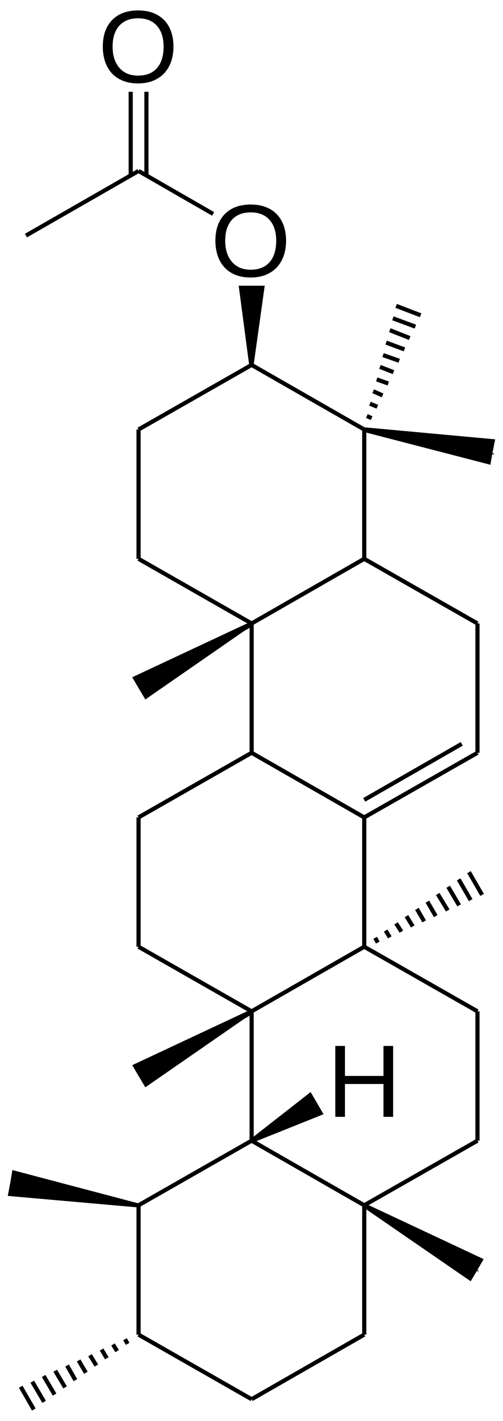

Bauer-7-en-3β-yl acetate

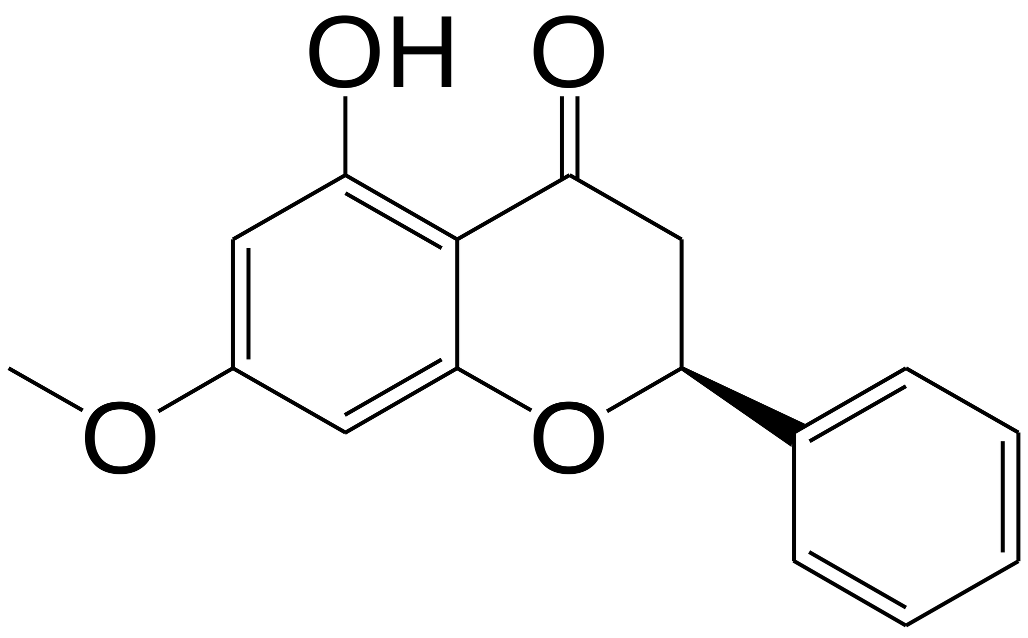

3,5-Dicaffeoylquinic acid

Supplementary Figure 1: Antiviral compounds of propolis (continued)

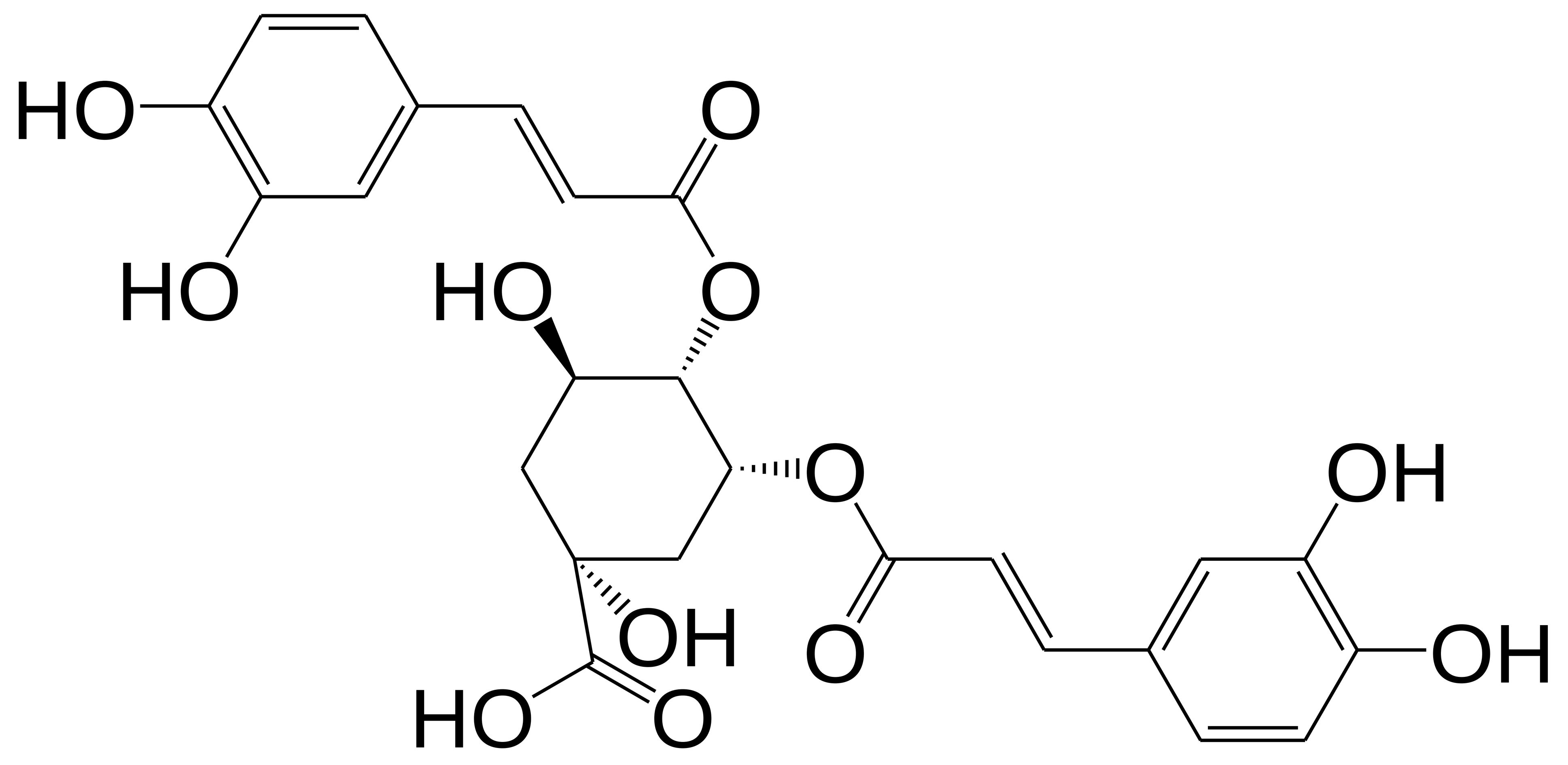

4,5-Dicaffeoylquinic acid

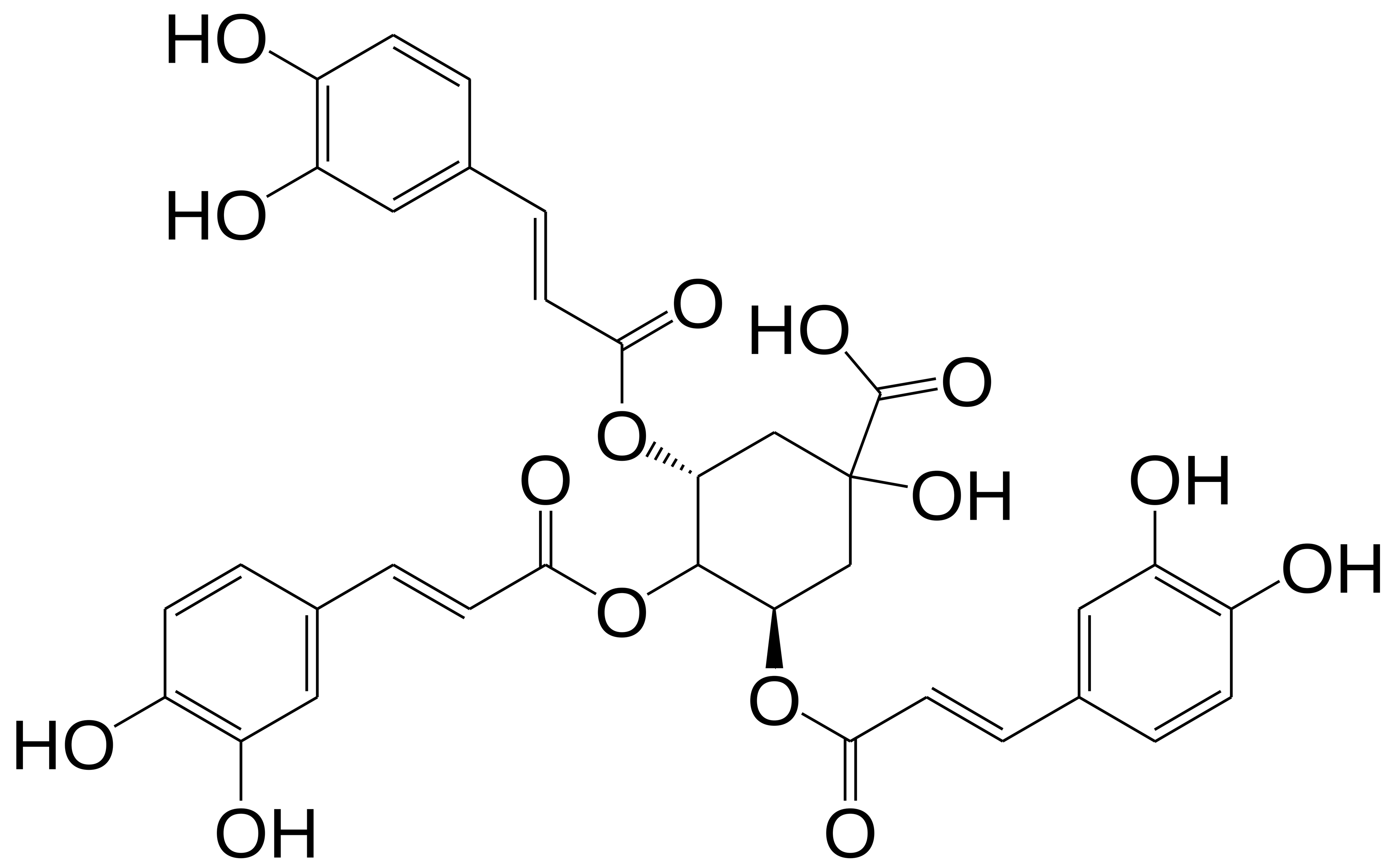

3,4,5-Tricaffeoylquinic acid

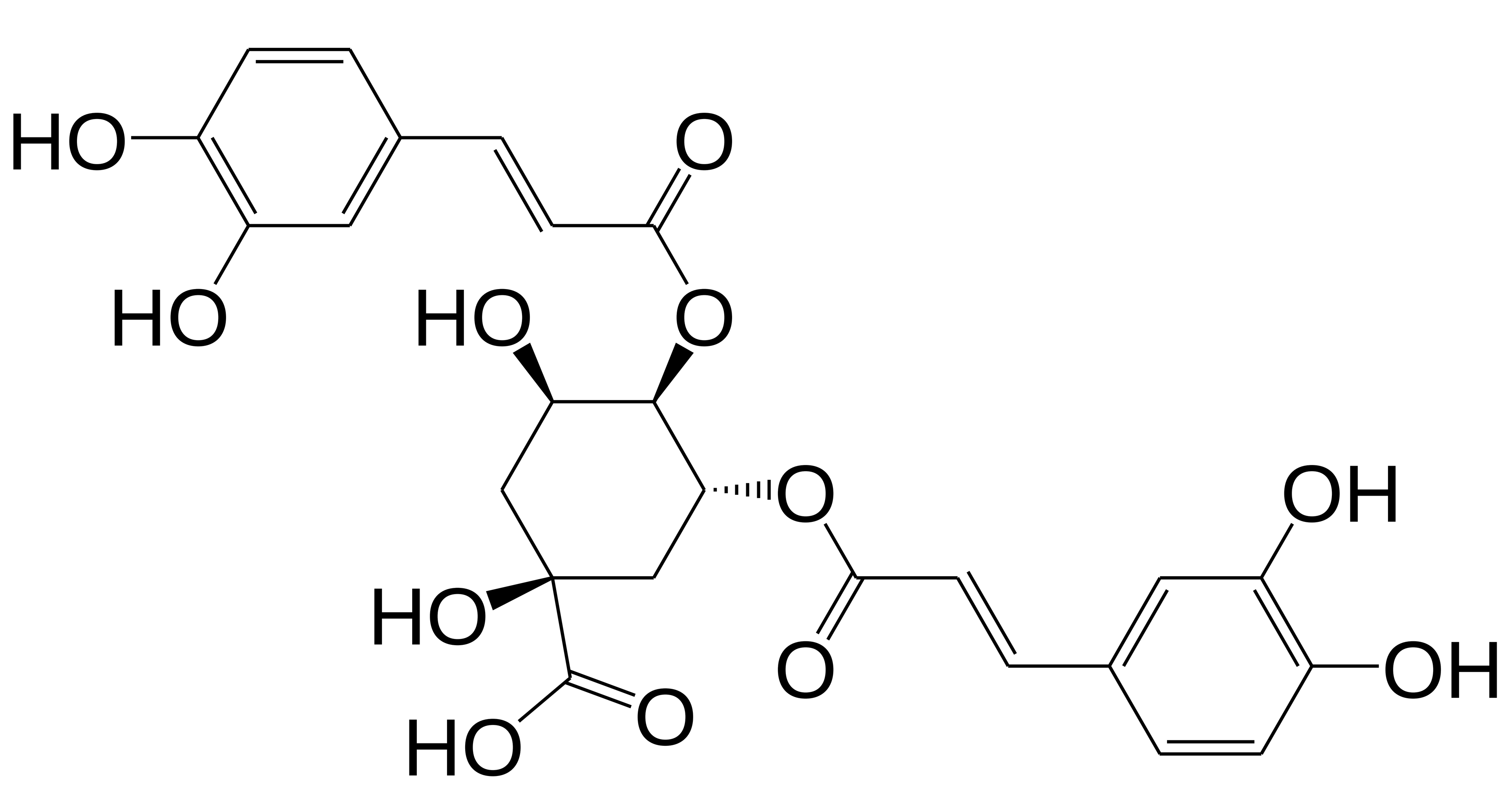

3,4-Dicaffeoylquinic acid

Supplementary Figure 1: Antiviral compounds in propolis
